# Effect of Thiol Molecular Structure on the Sensitivity of Gold Nanoparticle-Based Chemiresistors toward Carbonyl Compounds

**DOI:** 10.3390/s20247024

**Published:** 2020-12-08

**Authors:** Zhenzhen Xie, Mandapati V. Ramakrishnam Raju, Prasadanie K. Adhihetty, Xiao-An Fu, Michael H. Nantz

**Affiliations:** 1 Department of Chemical Engineering, University of Louisville, Louisville, KY 40208, USA; zhenzhen.xie@louisville.edu (Z.X.); xiaoan.fu@louisville.edu (X.-A.F.); 2 Department of Chemistry, University of Minnesota, Minneapolis, MN 55455, USA; smandapa@umn.edu; 3 Department of Chemistry, University of Louisville, Louisville, KY 40208, USA; prasadi.adhihetty@louisville.edu

**Keywords:** molecular selectivity, carbonyl sensing, urea, alkoxy urea, bis(alkoxy) urea

## Abstract

Increasing both the sensitivity and selectivity of thiol-functionalized gold nanoparticle chemiresistors remains a challenging issue in the quest to develop real-time gas sensors. The effects of thiol molecular structure on such sensor properties are not well understood. This study investigates the effects of steric as well as electronic effects in a panel of substituted thiol-urea compounds on the sensing properties of thiolate monolayer-protected gold nanoparticle chemiresistors. Three series of urea-substituted thiols with different peripheral end groups were synthesized for the study and used to prepare gold nanoparticle-based chemiresistors. The responses of the prepared sensors to trace volatile analytes were significantly affected by the urea functional motifs. The largest response for sensing acetone among the three series was observed for the thiol-urea sensor featuring a tert-butyl end group. Furthermore, the ligands fitted with N, N’-dialkyl urea moieties exhibit a much larger response to carbonyl analytes than the more acidic urea series containing N-alkoxy-N’-alkyl urea and N, N’-dialkoxy urea groups with the same peripheral end groups. The results show that the peripheral molecular structure of thiolate-coated gold nanoparticles plays a critical role in sensing target analytes.

## 1. Introduction

Monolayer protected gold clusters (MPCs) have attracted wide attention due to their unique electronic, electrochemical and biochemical properties [1,2,3,4,5] as well as their broad applications in materials science and biomedical diagnostics, where MPCs have been used as sensors [6,7,8], catalysts [9,10], biological imaging agents [11], and in studies on optics [12]. Many materials, including metal oxide nanoparticles and nanowires [13,14,15,16,17] and composites [18,19,20], semiconductors [21], carbon nanotubes [22,23], and polymer nanofibers [24,25], have been studied for the detection of a variety of volatile organic compounds (VOCs). However, these materials have encountered several challenges, including poor sensitivity and selectivity, lack of reproducibility, and large power consumption. Continued development of MPCs fitted with novel surface functionality has the potential to overcome these problems. The important characteristics of MPCs include operation at ambient temperature, variability in functional thiolate ligands, and the radial nature of the ligand monolayer arising from the faceted surface of the metal core [3,26,27].

Since Wohltjen and Snow reported the first chemiresistors of thiol-functionalized gold nanoparticles (AuNPs) for VOC detection [6], many researchers have investigated AuNP-based gas sensors for detecting various VOCs [28,29,30,31,32]. Unfortunately, high sensitivity and selectivity of AuNP chemiresistors toward sensing target analytes such as formaldehyde and acetone have not been demonstrated [4]. Furthermore, the effect of thiol molecular structure on the sensitivity and selectivity of thiol-functionalized AuNPs sensors has not been widely studied due to the limited availability of custom thiols. Most of the published AuNP-based gas sensors were prepared using commercial thiol-functionalized AuNPs [28,29,30,31,32].

The objective of this work is to identify new AuNP ligands that can significantly increase both sensitivity and selectivity for sensing carbonyl compounds by forming strong hydrogen-bonds with the carbonyl group of target analytes, such as formaldehyde, acetaldehyde and acetone. Formaldehyde, acetaldehyde and acetone are ubiquitous in environmental air. Chronic exposure to high concentrations of formaldehyde, acetaldehyde and acrolein can cause lung cancer and cardiovascular disease [33]. These aldehydes in the atmosphere are continuously monitored by the United States Environmental Protection Agency (EPA) under the National Air Toxics Assessment (NATA) program [34]. We sought to promote hydrogen-bond formation by incorporation of urea moieties on the surfaces of AuNPs for the detection of these carbonyl compounds in air. Hydrogen bonding has been used previously to promote crystal structures of adducts between urea and acetone derivatives [35]. We recently disclosed a synthesis of the urea-substituted thiol ligand 1-(tert-butyl)-3-((11-mercapto-undecyl)oxy)urea and its use in a AuNP-based sensor for detecting acetone in air [36]. We now seek to study the effects of peripheral end groups of this class of thiolate ligand to better understand how modulation of steric and electronic interactions influences chemiresistor sensitivity and selectivity. Steric interactions between the peripheral end groups can have significant effects on surface packing and thus on the stability and electronic behavior of AuNPs [37]. In the present work, three series of thiol-urea ligands were synthesized and then tested as sensing materials by incorporation onto AuNP chemiresistors. We report herein the results of acetone sensing studies and trends observed on varying the urea structure as well as peripheral groups.

## 2. Materials and Methods

### 2.1. Materials

**Scheme 1:**
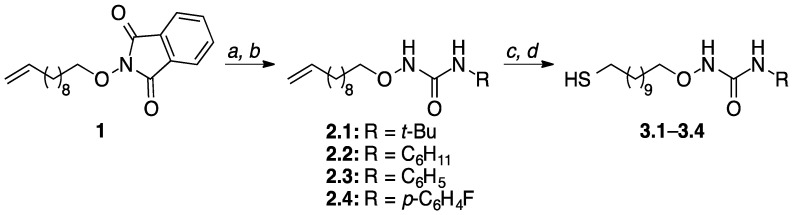
Synthesis of N-alkoxy-N’-alkylurea thiols 3.1–3.4 (Series I). Reagents and conditions: a. H_2_NNH_2_, THF, CH_2_Cl_2_, 0 °C to r.t.; b. R–NCO, Et_3_N, CH_2_Cl_2_, 0 °C to r.t.; c. CH_3_C(O)SH, cat. AIBN, THF, reflux; d. HCl, EtOH, reflux. AIBN = azoisobutyronitrile.

**Scheme 2:**
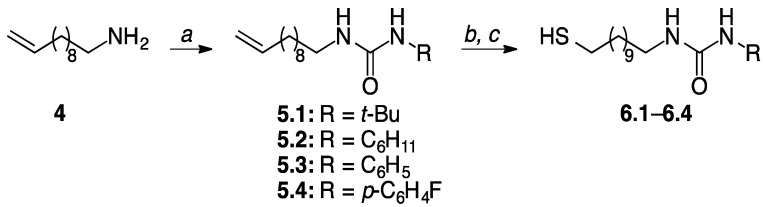
Synthesis of N,N’-dialkylurea thiols 6.1–6.4 (Series II). Reagents and conditions: a. R–NCO, Et_3_N, CH_2_Cl_2_, 0 °C to r.t.; b. CH_3_C(O)SH, cat. AIBN, THF, reflux; c. HCl, EtOH, reflux.

11-Bromo-1-undecene, thioacetic acid, and tert-butyl isocyanate were purchased from Sigma-Aldrich (St. Louis, MO, USA). N-Alkoxyphthalimide 1 (Scheme 1) was synthesized according to a published procedure [38]. Amine 4 (Scheme 2) and 1-(tert-butyl)-3-((11-mercaptoundecyl)-oxy)urea were prepared according to literature methods [39,40]. Hydrogen tetrachloroaurate (HAuCl_4_) and tetraoctylammonium bromide (TOAB) were obtained from Sigma-Aldrich. Sodium borohydride was purchased from Fluka (Buchs, Switzerland). Tedlar bags were purchased from Supelco (Bellefonte, PA, USA). Synthetic air with less than 4 ppm of moisture was obtained from a local gas supply company. Deionized water was used throughout this work.

Tetrahydrofuran (THF) was dried by distillation over Na/benzophenone. Dichloromethane and dimethylformamide (DMF) were dried by distillation over CaH_2_. Thin-layer chromatography using silica gel 60 A° F-254 plates was used to monitor the progress of reactions. The plates were visualized first by UV illumination and then by staining using a p-anisaldehyde stain. Column chromatography was performed using silica gel (230–400 mesh).

### 2.2. Thiol Ligand Syntheses

**Scheme 3:**
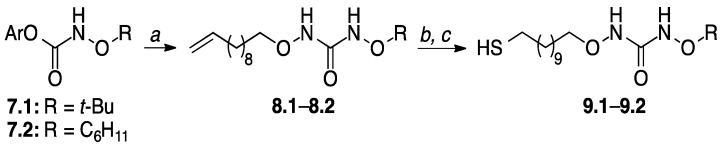
Synthesis of N,N’-dialkoxyurea thiols 9.1–9.2 (Series III). Reagents and conditions: a. H_2_C=CH(CH_2_)_9_ONH_2_ (fr. 1), Et_3_N, CH_2_Cl_2_, 0 °C to r.t.; b. CH_3_C(O)SH, cat. AIBN, THF, reflux; c. HCl, EtOH, reflux. Ar = p-C_6_H_4_NO_2_.

Syntheses of the three series of thiol-urea ligands with the general structure I, II and III in Table 1 were accomplished according to Scheme 1, Scheme 2 and Scheme 3 using routes analogous to the reported synthesis of 1-(tert-butyl)-3-((11-mercaptoundecyl)oxy)urea (3.1, Scheme 1) [36]. Step-by-step procedures for the synthesis of all ligands as well as all compound characterizations are provided in the Supporting Information (SI). All intermediate compounds and the final thiol ligands were analyzed and confirmed by ^1^H and ^13^C NMR spectroscopy. Exact masses of these compounds were obtained on a hybrid linear ion trap (LIT) FT-ICR mass spectrometer.

### 2.3. Thiol-Functionalized AuNP Synthesis

The Series I-III thiol-urea ligands in Table 1 were used as capping agents in the two-phase reduction approach developed by Brust et al. [41] to yield monolayer-protected gold nanoparticles that then were purified by precipitation from ethanol. A brief description of the method is presented here and follows the synthesis of 1-(tert-butyl)-3-(11-mercaptoundecyl)urea-functionalized AuNPs [36]. A solution containing HAuCl_4_ (0.05 g) dissolved in deionized water (4 mL) and another solution containing TOAB (0.08 g) in toluene (920 mL) were prepared. The HAuCl_4_ solution was then added into the solution of TOAB with vigorous stirring until all the HAuCl_4_ solution had transferred into the toluene solution. Each thiol of the three Series in Table 1 at a thiol:Au molar ratio of 1:1 was then added to the reaction mixture. Then, a freshly prepared aqueous solution of NaBH_4_ (0.056 g) in deionized water (4 mL) was slowly added to the mixture with vigorous stirring. There was a rapid color change on NaBH_4_ addition. After reaction, the organic phase was separated and the AuNPs were precipitated by dropwise addition of the organic phase into ethanol (400 mL) with rapid stirring. The average diameter of the thiol-coated AuNPs prepared in this way was about 2 nm as obtained from transmission electron microscopy images [36].

### 2.4. Fabrication of Interdigitated Electrodes and Thiol-Coated AuNP Chemiresistors

The detailed process of the fabrication of platinum interdigitated electrodes (IDE) has been reported [36]. The fabrication of thiol-coated AuNP sensors is briefly described here. AuNPs functionalized by all thiol ureas in Table 1 were dispersed in toluene (0.2% *w*/*w*) by sonication at ambient temperature. Then, the AuNPs dispersions were dropwise cast onto the platinum IDE area. Roughly circular films of thiol-functionalized AuNPs with a thickness of about 200 nm were formed after evaporation of toluene at ambient temperature. The resultant films were dried overnight at 40 °C in a vacuum oven. After the film preparation, the sensors of AuNPs coated with the three series of thiol-urea ligands were characterized for sensing target analytes in air to compare sensitivity and selectivity.

### 2.5. Sensor Measurements

All AuNP sensors functionalized with the Series I-III thiol-urea ligands for sensing target analytes were measured in the same way for direct comparison of the responses in order to understand the effect of thiol-urea molecular structure on sensing properties. The sensors were placed inside a stainless steel test chamber with a total volume of about 300 mL. The chamber was initially evacuated and VOC samples with known concentrations of analytes were introduced from a sample bag connected to the chamber. The pressure inside the chamber increased to the atmospheric pressure within a few seconds after the VOC sample entered into the chamber. During the sensing measurement, there was no air flow through the test chamber and the pressure inside the chamber was the atmospheric pressure. After testing the sample for a fixed time (e.g., 5 min), the chamber was evacuated for the next cycle of measurement.

VOC samples were prepared using Tedlar bags that had been washed with synthetic air three times. A calculated amount analyte was injected into a Tedlar bag containing 1L synthetic air for a given concentration. The concentrations of acetone in synthetic air samples were verified by a microreactor capture method as previously reported [42]. The sensors responded to VOCs with different concentrations in synthetic air by changing the resistance of the thiol-functionalized AuNP thin films on the IDE. The resistance was measured at an applied voltage using a Keithley 2400 I-V meter. All resistance data were recorded as a function of time using the Labview program. The voltage was fixed at 5 V and the current through the sensor was measured every second for calculation of the film resistance by Ohm’s law. All sensor resistances were first measured over 5 min in a vacuum of 28 inch Hg below atmospheric pressure, followed by VOC sample exposure at atmospheric pressure for 5 min, and then evacuation of the testing chamber. This measurement cycle of the sensor resistance in vacuum and VOC sample exposure was repeated at least three times for all samples. Three sensors of each thiol-functionalized AuNPs were tested for all acetone concentrations in synthetic air samples. Each sensor was examined from 10 ppb to 10 ppm of acetone in synthetic air. The responses are given as the average values of three different sensors.

## 3. Results and Discussion

**Figure 1:**
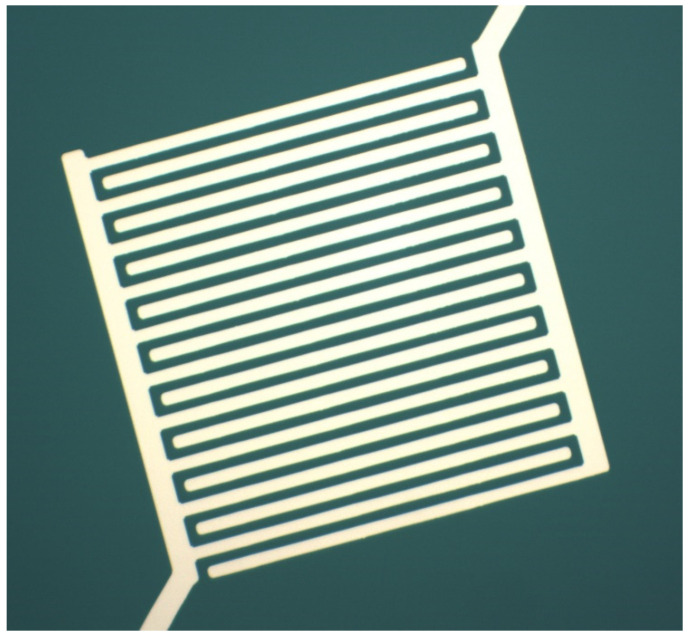
Microfabricated Pt/Ti platinum interdigitated electrodes (IDE) with 10 μm of both the width of the electrodes and the space between electrodes.

Figure 1 shows a typical optical microscope picture of the microfabricated IDE. All IDE have the same area of 400 μm × 400 μm and both the width of the electrodes and spaces between electrodes are 10 μm. The electrodes were made by sputtering 10-nm-thick titanium as an adhesion layer on a silicon dioxide insulating layer and a platinum layer (200 nm thick) on the top of the titanium layer. Thiol-functionalized AuNPs dispersed in toluene were droplet cast on the IDE to form sensors.

The AuNP sensors derived from Series I (3.1–3.4), Series II (6.1–6.4), and Series III (9.1–9.2) shown in Table 1 were compared for sensing acetone in synthetic air. The resistance of all sensors decreased for acetone in synthetic air in comparison with the resistance in synthetic air. Therefore, the sensor response to acetone in synthetic air is defined by the following equation according to the literature for sensing target analytes with decreased resistance [15,16]:

Response = (R_o_ − R_gas_)/R_gas_ (1)

where R_o_ and R_gas_ are the resistances of the sensor in synthetic air and in the presence of the acetone samples, respectively.

**Figure 2:**
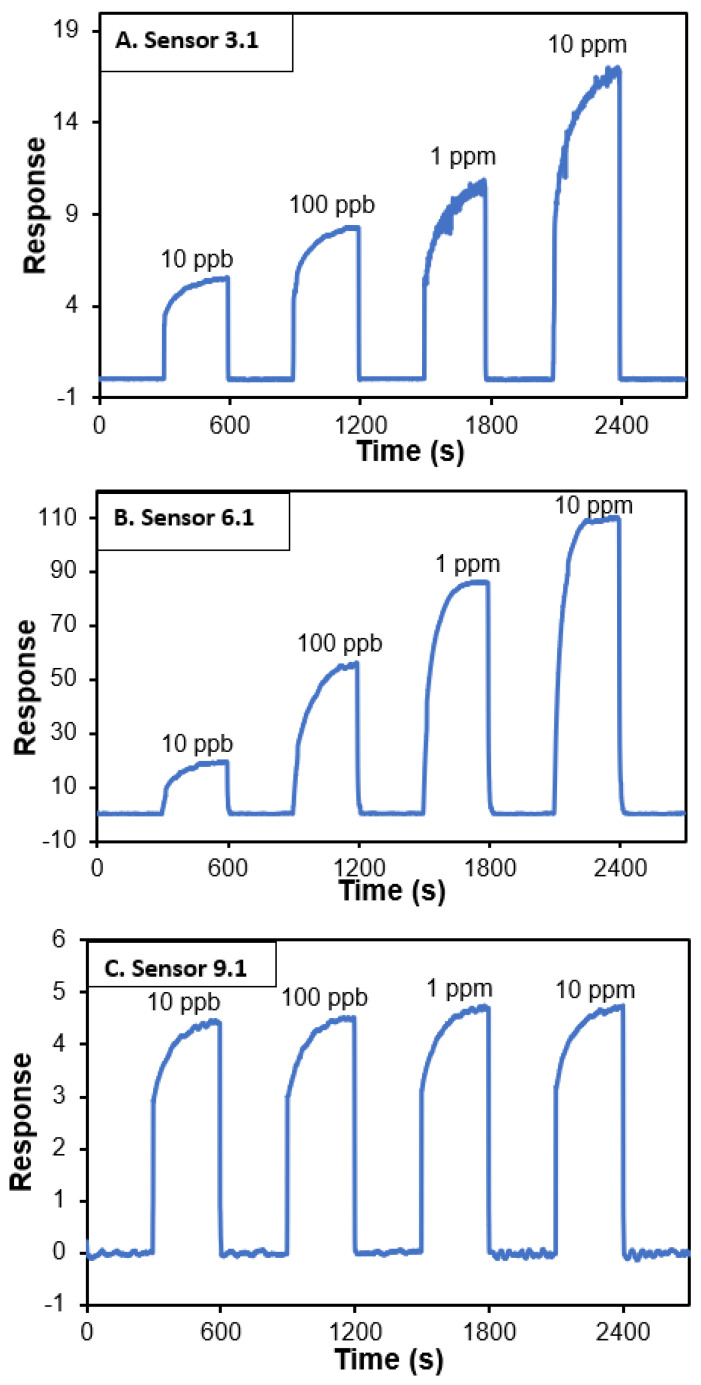
Responses of gold nanoparticle (AuNP) sensors derived from (**A**) N-alkoxy-N’-t-butyl urea 3.1, (**B**) N-alkyl-N’-t-butyl urea 6.1, and (**C**) N-alkoxy-N’-t-butoxy urea 9.1 for sensing acetone from 10 ppb to 10 ppm.

Figure 2 shows typical responses of the AuNP sensors derived from the three t-butyl analogs (3.1, 6.1, 9.1) over a wide concentration range of acetone, from 10 ppb to 10 ppm in synthetic air. For all tested concentrations, the AuNP sensors were sensitive toward acetone concentrations when coated with the alkoxy alkyl (Series I) thiol-urea 3.1 (Figure 2A) and dialkyl (Series II) thiol-urea 6.1 (Figure 2B), but not sensitive when using the dialkoxy (Series III) thiol-urea 9.1 (Figure 2C). The Series II t-butyl analog exhibited the highest response for all acetone concentrations that were studied. The N-alkyl-N’-t-butylurea-functionalized AuNPs sensor has both the highest sensitivity and lowest detection limit. Thus, the dialkyl thiol-urea 6.1 functionalized AuNP sensor provides higher responses than the alkoxy alkyl urea 3.1 and bisalkoxy urea 9.1 functionalized AuNP sensor.

**Figure 3:**
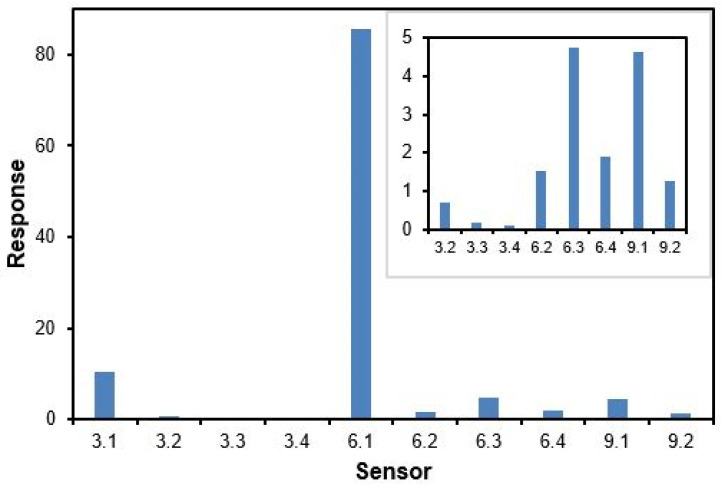
Response patterns for ten sensors functionalized with thiol-ureas (3.1–3.4 of Series 1, 6.1–6.4 of Series II and 9.1–9.2 of Series III) listed in Table 1 for sensing 1 ppm acetone in synthetic air. The inserted graph is a magnified view of all responses below 6.

**Figure 4:**
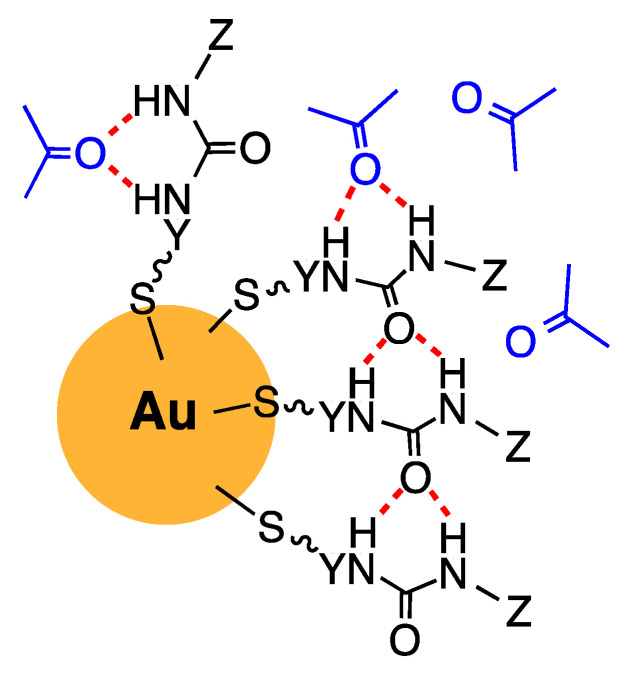
Model of H-bonding interactions between thiol-urea ligands (Series I, Y = O; II, Y = CH_2_; III, Y = O) and acetone at the surface of AuNPs where Z = peripheral alkyl, aryl, or alkoxy group.

To understand steric effects of the thiol-urea ligands and interactions between urea and the target analyte, we tested the response of AuNP sensors derived from all 10 thiol-ureas (Table 1) for sensing acetone (Figure 3). The inset of Figure 3 depicts a magnified view of the sensor responses below 6. We ascribe the larger responses observed for the t-butyl-substituted thiol-urea sensors (3.1, 6.1, 9.1), relative to the other peripherally substituted sensors within a given series, to a steric effect as well as coupled electronic interactions. We postulate that the bulkier t-butyl group decreases the ability to form H-bonds between adjacent thiol-urea ligands due to increased steric interactions [43,44]. This prevents close self-assembly of the ligands on the AuNP surfaces, which consequently increases opportunities for H-bonding with acetone. The peripheral cyclohexyl, phenyl and fluorophenyl groups of the dialkyl urea and mono-alkoxy alkyl urea ligands, Series I and II, respectively, did not provide high responses and sensitivity. These results point toward controlling the extent of the H-bonding network between urea moieties so as to enable interactions with carbonyl analytes. In this instance, the t-butyl substituent is sufficiently bulky to disrupt H-bonding between thiol-urea ligands on the AuNP surfaces to afford acetone access the urea group of the thiol to form H-bonding as illustrated Figure 4. Indeed, the effects of peripheral end groups of thiol amides on self-assembly at the surface of AuNPs have been studied by solid-state infrared spectroscopy and the results indicated that t-butyl groups have a weak H-bonding network, whereas aromatic end groups enhance H-bonding through favorable π-stacking interactions [37]. The near planar conformations of the cyclohexyl group presumably do not provide a sufficient steric barrier to disrupt the ligand H-bonding network.

By comparing the responses of ligands from different Series that have the same peripheral groups (Figure 4, Z = t-butyl, cyclohexyl, phenyl and fluorophenyl), it is clear that the dialkyl urea functionality (Series II) affords sensors (Figure 3, sensors e-h) that exhibit higher responses than the corresponding sensors derived from ligands having alkoxy alkyl urea or bisalkoxy urea functionality. The higher responses for the dialkyl urea-based sensors may be explained in part by the absence of an α-effect (i.e., the presence of an electronegative atom adjacent to the urea NH) [45,46]. The presence of the adjacent oxygen in the Series I and III ligands influences the urea NH acidity. Considering the relative pKa’s of protonated aminooxy (RONH_3_^+^, 5) [47] vs. aminium (RNH_3_^+^, 10), it is reasonable to expect that both the alkoxy alkyl urea and dialkoxy urea will be more acidic than the dialkyl urea [48]. Higher urea acidity, in turn, will result in a more extensive H-bonding network between adjacent urea ligands. As intermolecular forces contribute to pack the ligand chains closer together, it becomes more difficult for carbonyl analytes to H-bond with the urea moiety and become associated with the AuNP surface. Thus, the weaker H-bonding network of the dialkyl urea motif and the steric destabilization afforded by the t-butyl end group, as in the sensor derived from ligand 6.1 in Scheme 2, combine to provide the highest sensor response for detection of acetone.

**Figure 5:**
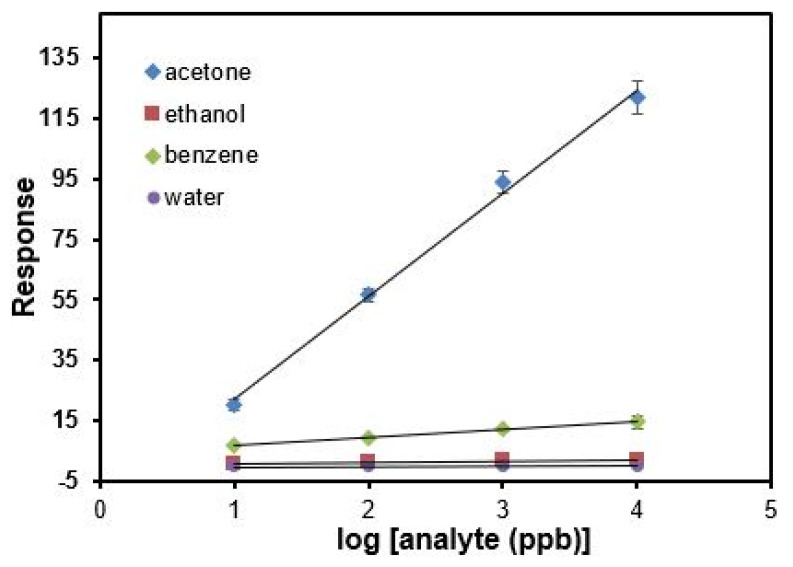
Response of a AuNP sensor derived from t-butyl urea 6.1 vs. log[analyte (ppb)] in the detection of acetone, ethanol and benzene.

Given the high response toward acetone when using the t-butyl substituted dialkyl urea 6.1, we further compared the acetone-sensing sensitivity and selectivity of this thiol urea functionalized AuNP sensor with its responses to ethanol, water and benzene. Figure 5 shows that the smaller slopes of the sensor response obtained for ethanol (0.39), water (0.35) and benzene (2.52) compared to that of acetone (33.2). The slope of the sensor’s linear response to the log function of the analytes is a direct measure of the sensitivity and selectivity. The results indicate that the sensor has much higher sensitivity and selectivity for acetone than ethanol, water and benzene. The response of the sensor to acetone is about 20 times of that to ethanol and water and seven times of that to benzene at the concentration of 1 ppm in synthetic air. The H-bonding-induced association of acetone with the urea group of the t-butyl substituted dialkyl urea 6.1 increased the sensitivity and selectivity of the sensor.

## 4. Conclusions

Thiol-urea ligand panels featuring alkoxy alkyl, dialkyl, and dialkoxy urea groups having different peripheral substituents were synthesized to examine the effect of molecular structures on gold nanoparticle-based gas sensing properties. The dialkyl urea-functionalized AuNP sensors (Series II) showed the higher sensitivities, among which the ligand having a t-butyl end group exhibited the highest sensitivity as well as selectivity toward detecting acetone. The results suggest that modulation of the intermolecular H-bonding network between ligand chains, a function of urea acidity and end group destabilization, is an important means for improving the sensitivity and possibly selectivity. The results also support the concept of using designed thiols for selective interaction with target analytes based on functional group interactions, such as urea-carbonyl H-bonding, to furnish chemoselective AuNP chemiresistors with enhanced sensitivity.

## Figures and Tables

**Table 1:** Panel of thiol-urea ligands synthesized for comparison.

Series I Alkoxy Alkyl (3.1–3.4)	Series II Dialkyl (6.1–6.4)	Series III Dialkoxy (9.1–9.2)
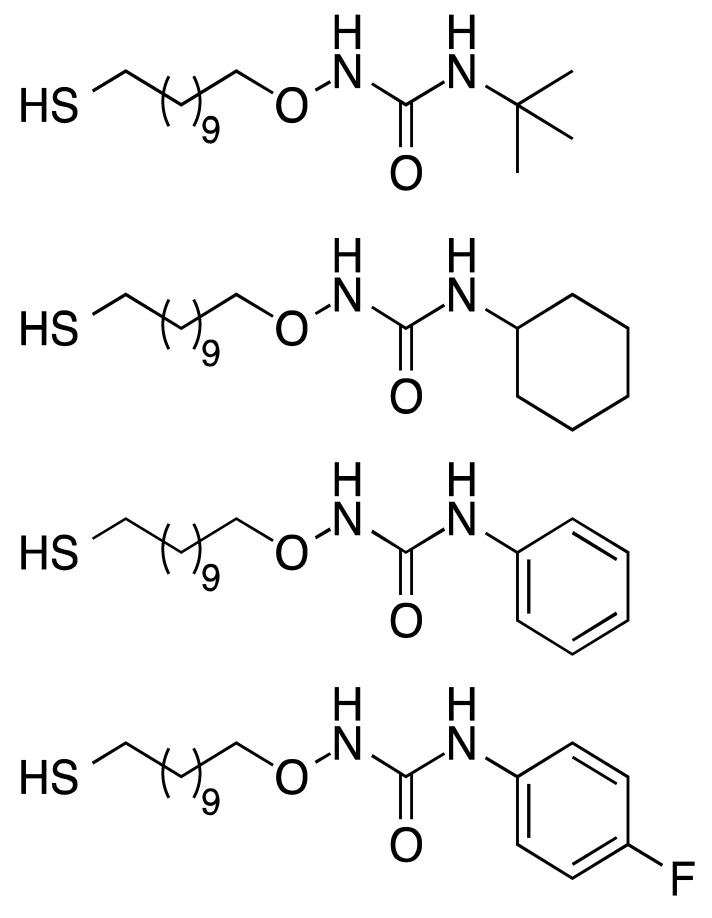	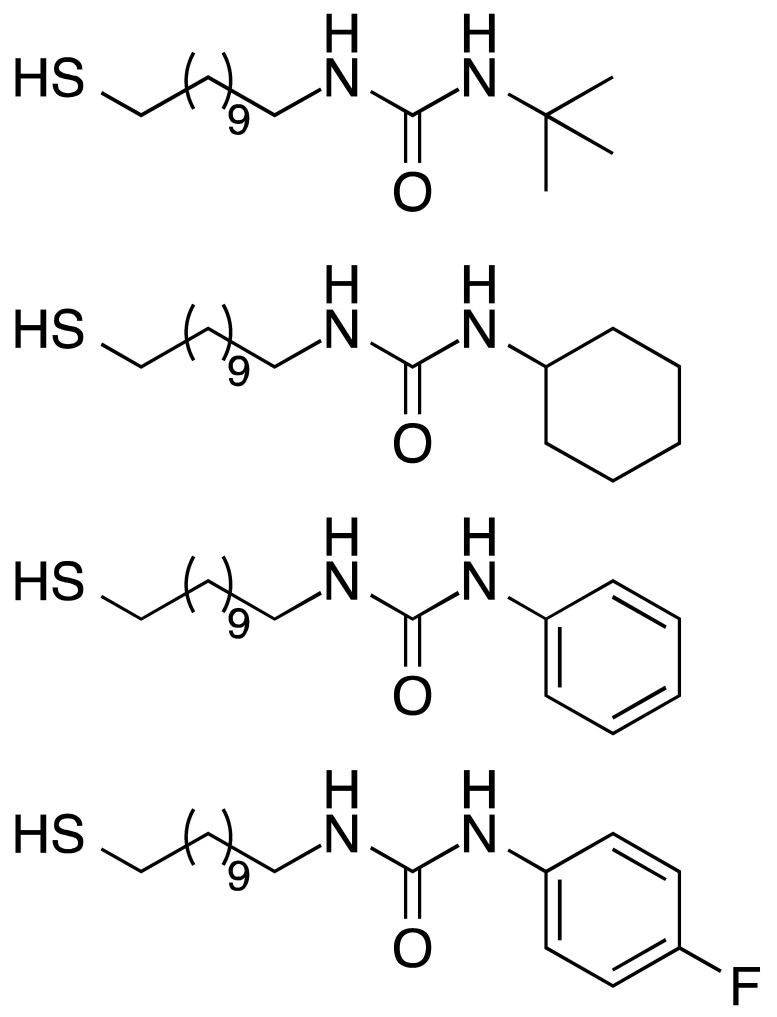	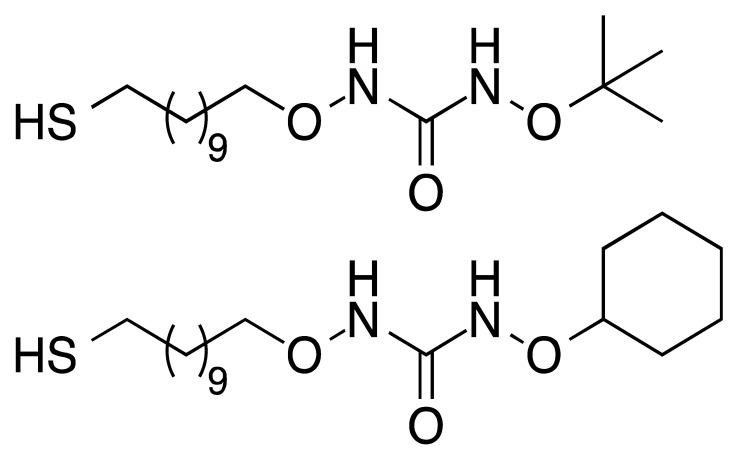

## Supplementary Materials

The following Materials and Analytical Methods, thiol synthesis procedures and ^1^H and ^13^C NMR Spectra are available online at https://www.mdpi.com/1424-8220/20/24/7024/s1.
