# Supplementary material for: Effect of Thiol Molecular Structure on the Sensitivity of Gold Nanoparticle-Based Chemiresistors toward Carbonyl Compounds
Source: Sensors (Basel). 2020 Dec 8;20(24):7024. doi: 10.3390/s20247024 (PMC7763667; doi:10.3390/s20247024)

Supplementary Information

to accompany the manuscript entitled

Effect of thiol molecular structure on the sensitivity of gold nanoparticle-based chemiresistors toward carbonyl compounds

Zhenzhen Xie, Mandapati V. Ramakrishnam Raju, Xiao-An Fu and Michael H. Nantz

Table of Contents

I. Materials and Analytical Methods S2

II. Experimental Procedures S3

III. ^{1}H and ^{13}C NMR Spectra S14

I. Materials and Analytical Methods

Reagent grade solvents were used for extractions and for purifications via flash column chromatography. THF was dried by distillation over Na/benzophenone. DCM and DMF were dried by distillation over CaH_2.

The progress of reactions was monitored by thin-layer chromatography using silica gel 60 A° F-254 plates. The plates were visualized first by UV illumination and then by staining using a *p*-anisaldehyde stain. Column chromatography was performed using silica gel (230-400 mesh).

11-Bromo-1-undecene, thioacetic acid, and *tert*-butyl isocyanate were purchased from Sigma-Aldrich. *N*-Alkoxypthalimide **2** (Scheme S1) was prepared according to a literature procedure.[1] 1-(*tert*-Butyl)-3-((11-mercapto-undecyl)oxy)urea (**1**) was prepared by analogy to a reported method.[2] Hydrogen tetrachloroaurate ($HAuCl_4$), 1-dodecanethiol ($HS-(CH_2)_{11}-CH_3$), 4-methoxy-α-toluenethiol ($HS-C_6H_6-OCH_3$), 11-mercaptoundecanoic acid ($HS-(CH_2)_{10}-COOH$, 11-MUA), tetraoctylammonium bromide (TOAB) and Tedlar bags also were obtained from Sigma-Aldrich Chemical Co. Sodium borohydride was purchased from Fluka. Deionized water was used throughout all experimentation.

NMR spectra were obtained using a Varian/Agilent 400-MHz NMR spectrometer equipped with a 5 mm z-axis gradient AutoX probe operating at the nominal ^{1}H frequency of 399.66 MHz and ^{13}C frequency of 100.49 MHz. All spectra are reported in parts per million (ppm) relative to the residual solvent peak in ^{1}H NMR and the deuterated solvent peak in ^{13}C NMR. Mass samples were analyzed on a hybrid linear ion trap (LIT) FT-ICR mass spectrometer (LTQ-FT, Thermo Electron, Bremen, Germany) equipped with a TriVersa NanoMate ion source (Advion BioSciences, Ithaca, NY) with an ‘‘A’’electrospray chip (nozzle inner diameter 5.5 lm). The TriVersa NanoMate was operated in positive ion mode by applying 1.85 kV with 0.1 psi head pressure. Melting points were acquired using Fisher-Johns melting point apparatus.

II. Experimental Procedures

Series I synthesis procedures

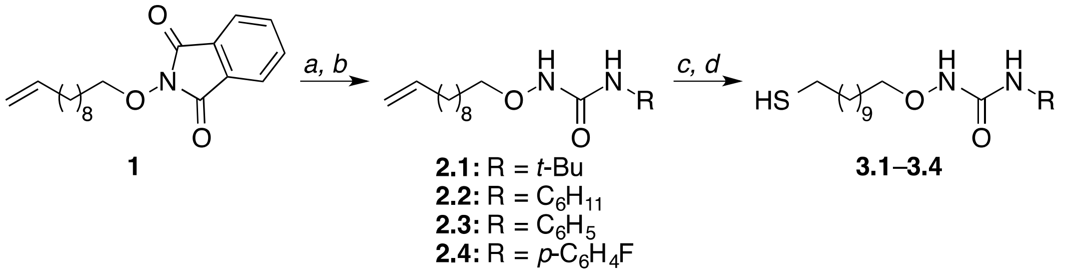

Scheme S1. Synthesis of *N*-alkoxy-*N'*-alkylurea thiols **3.1-3.4** (Series I). Reagents and conditions: *a*. H_2NNH_2, THF, CH_2Cl_2, 0 °C to rt; *b*. R–NCO, Et_3N, CH_2Cl_2, 0 °C to rt; *c*. $CH_3C(O)SH$, cat. AIBN, THF, reflux; *d*. HCl, EtOH, reflux. AIBN = azoisobutyronitrile

Hydrazinolysis (step *a*, Scheme S1). To a solution of phthalimide **1** (1.00 g, 3.17 mmol) in CH_2Cl_2 (10 mL) at 0 °C was added hydrazine (10.2 mL of 1.0 M solution in THF, 10.2 mmol). The reaction mixture was stirred at 0 °C for 1.5h and then filtered. The retentate was washed with CH_2Cl_2 (10 mL) and the combined organic solvents then were concentrated by rotary evaporation. The crude aminooxy product so obtained was used directly in the next step without further purification.

Intermediate urea formations (step b, Scheme S1)

1-(*tert*-Butyl)-3-(undec-10-en-1-yloxy)urea (**2.1**). To a stirred solution of the crude aminooxy (0.58 g, 3.17 mmol) in dry CH_2Cl_2 (15 mL) at room temperature under argon was added dry Et_3N (0.48 mL, 3.45 mmol) via syringe. The reaction mixture was cooled to 0 °C and then *t*-butyl isocyanate (0.32 mL, 2.87 mmol) was added dropwise over 5 min. The reaction mixture was slowly allowed to reach room temperature and stirred 10 h before concentrating by rotary evaporation. The residue was purified by SiO_2 flash column chromatography, eluting with a 1:4 mixture of EtOAc:hexane, to afford alkoxyurea **2.1** (0.62 g, 68%) as a colorless liquid; TLC, R_f = 0.51 (2.5:7.5, EtOAc:hexane); IR 3422, 3194, 2923, 2855, 1674, 1525 cm^{-1};^{1}H NMR ($CDCl_3$, 400 MHz) δ 6.85 (br, NH), 5.84-5.74 (m, 1H), 5.59 (br, NH), 5.00-4.90 (m, 2H), 3.77 (t, *J* = 6.4 Hz, 2H), 2.05-2.00 (m, 2H), 1.64-1.57 (m, 2H), 1.36-1.27 (m, 21H) ppm; ^{13}C NMR ($CDCl_3$, 100 MHz) δ 159.3, 139.1, 114.2, 76 .4, 50.4, 33.8, 29.5, 29.4, 29.18, 29.12, 28.9, 28.1, 26.1 ppm.

1-Cyclohexyl-3-(undec-10-en-1-yloxy)urea (**2.2**). Using the general procedures outlined above for synthesis of **2.1,** phthalimide **1** (0.51 g, 1.61 mmol) was transformed into **2.2** (0.36 g, 71%) as a white solid; TLC, R_f = 0.44 (2.5:7.5, EtOAc:hexane); IR 3226, 3201, 2921, 2852, 1639 cm^{-1}; ^{1}H NMR ($CDCl_3$, 400 MHz) δ 6.77 (br, NH), 5.84-5.76 (m, 1H), 5.59 (d, J = 8.0 Hz, 1H), 5.01-4.92 (m, 2H), 3.79 (t, J = 6.8 Hz, 2H), 3.67 (t, J = 4.0 Hz, 1H), 2.06-2.01 (m, 2H), 1.97-1.93 (m, 2H), 1.73-1.69 (m, 2H), 1.68-1.56 (m, 3H), 1.44-1.29 (m, 14H), 1.23-1.14 (m, 3H) ppm; ^{13}C NMR ($CDCl_3$, 100 MHz) δ 159.5, 139.2, 114.2, 76.7, 48.4, 33.9, 33.6, 29.6, 29.5, 29.2, 29.0, 28.2, 26.2, 25.6, 24.9 ppm.

1-Phenyl-3-(undec-10-en-1-yloxy)urea (**2.3**). Using the general procedures outlined above for synthesis of **2.1**, phthalimide **1** (0.30 g, 0.95 mmol) was transformed into **2.3** (0.22 g, 77%) as a white solid; IR 3332, 3172, 2915, 2849, 1655 cm^{-1}; ^{1}H NMR ($CDCl_3$, 400 MHz) δ 7.56 (br, NH), 7.47 (d, J = 8.0 Hz, 2H), 7.32 (t, J = 8.0 Hz, 2H), 7.09 (t, J = 7.8 Hz, 1H), 5.84-5.78 (m, 1H), 5.01-4.92 (m, 2H), 3.91(t, J = 6.4 Hz, 2H), 2.06-2.01 (m, 2H), 1.74-1.67 (m, 2H), 1.39-1.29 (m, 12H) ppm; ^{13}C NMR ($CDCl_3$, 100 MHz) δ 157.3, 139.2, 137.4, 129.2, 124.0, 119.6, 114.3, 77.4, 33.9, 29.6, 29.5, 29.2, 29.0, 28.2, 26.1 ppm.

1-(4-Fluorophenyl)-3-(undec-10-en-1-yloxy)urea (**2.4**). Using the general procedures outlined above for synthesis of **2.1**, phthalimide **1** (0.52 g, 1.64 mmol) was transformed into **2.4** (0.35 g, 66%) as a white solid; IR 3328, 3211, 2922, 2854, 1659 cm^{-1}; ^{1}H NMR ($CDCl_3$, 400 MHz) δ 7.51 (br, 1H), 7.44-7.40 (m 2H), , 7.04-7.00 (m, 2H), 5.84-5.78 (m, 1H), 5.01-4.92 (m, 2H), 3.91 (t, J = 6.4 Hz, 2H), 2.06-2.-01 (m, 2H), 1.74-1.70 (m, 2H), 1.39-1.29 (m, 12H) ppm; ^{13}C NMR ($CDCl_3$, 100 MHz) δ 160.5, 158.1, 157.5, 139.2, 133.4, 121.5 (2), 115.8 (2), 114.2, 77.4, 36.5, 33.8, 29.5, 29.4, 29.1, 29.0, 28.1, 26.0 ppm.

Intermediate thioester formations (step c, Scheme S1)

***S*-(11-((3-(*tert*-Butyl)ureido)oxy)undecyl) ethanethioate**. A solution of urea **2.1** (0.50 g, 1.75 mmol) and azobis(isobutyronitrile) (0.043 g, 0.26 mmol) in dry THF (20 mL) was heated to reflux whereupon thioacetic acid (0.25 mL, 3.51 mmol) was added via syringe. The reaction mixture was stirred at reflux 8h before concentrating by rotary evaporation. The residue was purified by SiO_2 column chromatography, eluting with a 15:85 mixture of EtOAc:hexane, to afford the title thioester (0.48 g, 75%) as a pale yellow liquid; ^{1}H NMR ($CDCl_3$, 400 MHz) δ

6.91 (br, NH), 5.59 (br, NH), 3.76 (t, J = 6.8 Hz, 2H), 2.84 (t, J = 6.8 Hz, 2H,), 2.30 (s, 3H), 1.59-1.50 (m, 4H), 1.35-1.25 (m, 23H), ppm; ^{13}C NMR ($CDCl_3$, 100 MHz) δ 196.1, 159.2, 76.4, 50.5, 30.7, 30.6, 29.5, 29.2, 28.8, 28.2, 26.1 ppm.

S-(11-((3-Cyclohexylureido)oxy)undecyl) ethanethioate. Using the general procedure outlined above for thioester formation, urea **2.2** (0.25 g, 0.80 mmol) was transformed into the title thioester (0.26 g, 83%) as a pale white solid; IR 3322, 3200, 2919, 2851, 1690, 1639, 1535 cm^{-1}; ^{1}H NMR ($CDCl_3$, 400 MHz) δ 6.83 (d, J = 5.6 Hz, NH), 5.57 (d, J = 7.6 Hz, NH) 3.79 (t, J = 6.4 Hz, 2H), 3.67 (t, J = 4.4 Hz, 1H), 2.85 (t, J = 7.6 Hz, 2H), 2.32 (s, 3H), 1.96-1.94 (m, 2H), 1.72-1.69 (m, 2H), 1.64-1.42 (m, 5H), 1.39-1.22(m, 16H), 1.19-1.14 (m, 3H) ppm; ^{13}C NMR ($CDCl_3$, 100 MHz) δ 196.1, 159.4, 76.6, 48.4, 48.3, 33.6, 30.8, 30.7, 29.6, 29.4, 29.2, 29.1, 28.9, 28.2, 26.1, 25.6, 24.9 ppm.

S-(11-((3-Phenylureido)oxy)undecyl) ethanethioate. Using the general procedure outlined above for thioester formation, urea **2.3** (0.20 g, 0.66 mmol) was transformed into the title thioester (0.26 g, 83%) as a white solid; IR 3363, 3195, 2922, 2848, 1681, 1655, 1596 cm^{-1}; ^{1}H NMR ($CDCl_3$, 400 MHz) δ 7.56 (br, NH), 7.48-7.45 (m, 2H), 7.34-7.30 (m, 2H), 7.09 (t, J = 7.2 Hz, 1H), 3.91 (t, J = 6.8 Hz, 2H), 2.85 (t, J = 7.6 Hz, 2H), 2.31 (s, 3H), 1.72-1.66 (m, 2H), 1.57-1.41 m, 2H), 1.40-1.27(m, 14H) ppm; ^{13}C NMR ($CDCl_3$, 100 MHz) δ 196.2, 157.3, 137.4, 129.1, 124.0, 119.6, 77.4, 30.8, 30.7, 29.5 x 2, 29.4, 29.2, 29.1, 28.9, 28.2, 26.1 ppm.

S-(11-((3-(4-Fluorophenyl)ureido)oxy)undecyl) ethanethioate. Using the general procedure outlined above for thioester formation, urea **2.4** (0.25 g, 0.28 mmol) was transformed into the title thioester (0.18 g, 58%) as a white solid; IR 3384, 3184, 2922, 2854, 1678, 1660, 1525 cm^{-1}; ^{1}H NMR ($CDCl_3$, 400 MHz) δ 7.51 (br, 1H), 7.44-7.40 (m, 2H), 7.04-7.00 (m, 3H), 3.91 (t, J = 6.8 Hz, 2H), 2.86 (t, J = 7.2 Hz, 2H), 2.32 (s, 3H), 1.72-1.66 (m, 2H), 1.57-1.52 (m, 2H), 1.39-1.27 (m, 14H) ppm; ^{13}C NMR ($CDCl_3$, 100 MHz) δ 196.2, 160.6, 158.2, 157.4, 133.4, 121.5, 115.9, 115.7, 77.4, 30.8, 30.7, 29.5 (2), 29.4, 29.2, 29.1, 28.9, 28.2, 26.0 ppm.

Intermediate thioester cleavages (step d, Scheme S1)

1-(*tert*-Butyl)-3-((11-mercaptoundecyl)oxy)urea (**3.1**). To a solution of the intermediate thioester prepared above (0.50 g, 1.38 mmol) in EtOH (10 mL) was added 12 N HCl (1.1 mL, 11.0 mmol). The reaction mixture was heated at reflux and stirred 4h, whereupon the reaction

mixture was cooled and then concentrated by rotary evaporation. The residue was dissolved in water (10 mL). The resultant solution was cooled to 0 °C and the pH was adjusted to slightly alkaline using saturated $NaHCO_3$ solution. The aqueous layer then was extracted with CH_2Cl_2 (4 X 30 mL). The combined organic phase was washed with saturated NaCl (2 X 20 mL), dried ($MgSO_4$), filtered and concentrated under reduced pressure to afford thiol-urea **3.1** (0.38 g, 86%) as a pale yellow liquid that was directly applied to gold nanoparticles; ITMS for $C_{16}H_{34}N_2O_2S$ calcd 319.2419, found 319. 2419 $[M+H]^+$; IR 3422, 3194, 2923, 2855, 1674, 1525, 1457, 1364, 1235 cm^{-1}; ^1H NMR ($CDCl_3$, 400 MHz) δ 6.76 (br s, 1H, NH), 5.60 (br s, 1H, NH), 3.77 (t, J = 6.8 Hz, 2H), 2.51 (q, J = 7.4 Hz, 2H), 1.61-1.55 (m, 4H), 1.36-1.26 (m, 23H, t-butyl + aliphatic chain) ppm; ^{13}C NMR ($CDCl_3$, 100 MHz) δ 159.2, 76.4, 50.4, 34.0, 29.5, 29.4, 29.2, 29.1, 28.3, 28.2, 26.1, 24.6 ppm.

1-Cyclohexyl-3-((11-mercaptoundecyl)oxy)urea (**3.2**). Using the general procedure outlined above for synthesis of thiol-urea **3.1**, the corresponding intermediate thioester (0.230 g, 0.57 mmol) was transformed into thiol-urea **3.2** (0.18 g, 88%) as a white solid that was directly applied to gold nanoparticles; IR 3322, 3205, 2919, 2850, 1639, 1536, 1334 cm^{-1}; ^1H NMR ($CDCl_3$, 400 MHz) δ 6.82 (d, J = 11.2 Hz, NH), 5.58 (d, J = 7.2 Hz, NH), 3.79 (t, J = 6.4 Hz, 2H), 3.70-3.63 (m, 1H), 2.54-2.49 (m, 2H), 1.96-1.93 (m, 2H), 1.72-1.69 (m, 2H), 1.64-1.56 (m, 5H), 1.44-1.22 (m, 16H), 1.19-1.14 (m, 3H) ppm; ^{13}C NMR ($CDCl_3$, 100 MHz) δ 159.5, 77.4, 48.4, 48.3, 34.1, 33.6, 29.6, 29.4, 29.1, 28.4, 28.2, 26.1, 25.6, 24.9, 24.7 ppm.

1-((11-Mercaptoundecyl)oxy)-3-phenylurea (**3.3**). Using the general procedure outlined above for synthesis of thiol-urea **3.1**, the corresponding intermediate thioester (0.175 g, 0.46 mmol) was transformed into thiol-urea **3.3** (0.120 g, 77%) as a white solid that was directly applied to gold nanoparticles; IR 3331, 3192, 2918, 2849, 1657, 1532 cm^{-1}; ^1H NMR ($CDCl_3$, 400 MHz) δ 7.56 (br, NH), 7.47-7.45 (m, 2H), 7.34-7.30 (m, 2H), 7.11-7.0 (m, 1H), 3.91 (t, J = 6.8 Hz, 2H), 2.52 (q, J = 7.2 Hz, 2H), 1.72-1.68 (m, 2H), 1.62-1.58 (m, 2H), 1.40-1.28 (m, 14H) ppm; ^{13}C NMR ($CDCl_3$, 100 MHz) δ 157.3, 137.4, 129.1, 124.0, 119.6, 76.8, 34.1, 29.5, 29.4, 29.1, 28.4, 28.2, 26.0, 24.7 ppm.

1-(4-Fluorophenyl)-3-((11-mercaptoundecyl)oxy)urea (**3.4**). Using the general procedure outlined above for synthesis of thiol-urea **3.1**, the corresponding intermediate thioester (0.155 g, 1.17 mmol) was transformed into thiol-urea **3.4** (0.110 g, 79%) as a pale white solid

that was directly applied to gold nanoparticles; IR 3367, 3206, 2917, 2854, 1657, 1524, 1509 cm^{-1}; ^{1}H NMR ($CDCl_3$, 400 MHz) δ 7.51 (br, NH), 7.43-7.41 (m, 2H), 7.09 (br, 1H), 7.04-7.00 (m, 2H), 3.91 (t, J = 6.4 Hz, 2H), 2.52 (q, J = 8.0 Hz, 2H), 1.73-1.68 (m, 2H), 1.66-1.60 (m, 2H), 1.38-1.28 (m, 14H) ppm; ^{13}C NMR ($CDCl_3$, 100 MHz) δ 160.5, 158.1, 157.5, 133.4, 121.6, 121.5, 115.9, 115.6, 77.4, 34.1, 29.5, 29.4, 29.1, 28.4, 28.2, 26.0, 24.7 ppm.

Series II synthesis procedures

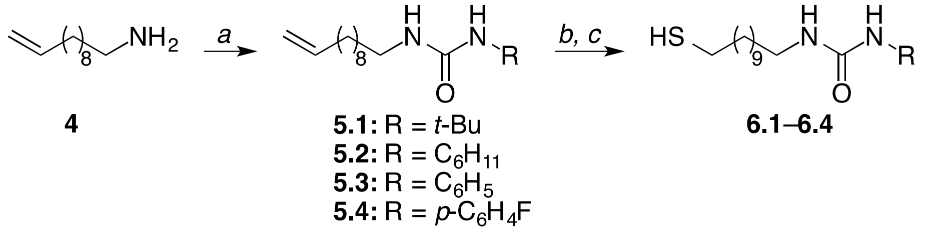

Scheme S2. Synthesis of *N,N'*-dialkylurea thiols **6.1-6.4** (Series II). Reagents and conditions: *a*. R–NCO, Et_3N, CH_2Cl_2, 0 °C to rt; *b*. $CH_3C(O)SH$, cat. AIBN, THF, reflux; *c*. HCl, EtOH, reflux.

Intermediate urea formations (step a, Scheme S2)

1-(*tert*-Butyl)-3-(undec-10-en-1-yl)urea (**5.1**). To a stirred solution of amine **4** (0.56 g, 3.32 mmol) in dry CH_2Cl_2 (15 mL) at rt under argon was added dry Et_3N (0.64 mL, 4.53 mmol) via syringe. The reaction mixture was cooled to 0 °C and then *t*-butyl isocyanate (0.34 mL, 3.02 mmol) was added dropwise over 5 min. The reaction mixture was slowly allowed to reach rt and then stirred 10h before concentrating by rotary evaporation. The residue was purified by SiO_2 flash column chromatography, eluting with a 1:4 mixture of EtOAc:hexane, to afford dialkylurea **5.1** (0.65 g, 72%) as a colorless liquid; IR 3321, 2971, 2922, 2852, 1738, 1630, 1562 cm^{-1}; ^{1}H NMR ($CDCl_3$, 400 MHz) δ 5.83-5.75 (m, 1H), 501-4.91 (m, 2H), 4.21-4.12 (br, 2H), 3.09 (t, J = 6.8 Hz, 2H), 2.05-2.00 (m, 2H), 1.47-1.43 (m, 2H), 1.38-1.26 (m, 21H) ppm; ^{13}C NMR ($CDCl_3$, 100 MHz) δ 157.8, 139.3 x 2, 114.4, 11.2, 50.4, 40.5, 33.9, 30.4, 29.7, 29.6, 29.5 x 2, 29.2, 29.0, 27.1 ppm.

1-Cyclohexyl-3-(undec-10-en-1-yl)urea (**5.2**). Using the general procedure outlined above for synthesis of urea **5.1**, amine **4** (0.79 g, 2.64 mmol) was transformed into urea **5.2** (0.58 g,

75%) as a white solid; IR 3322, 2923, 2851, 1622, 1574 cm^{-1}; ^1H NMR ($CDCl_3$, 400 MHz) δ 5.84-5.74 (m, 1H), 5.00-4.90 (m, 2H), 3.77 (t, J = 6.4 Hz, 2H), 2.05-2.00 (m, 2H), 1.64-1.57 (m, 2H), 1.36-1.27 (m, 21H) ppm; ^{13}C NMR ($CDCl_3$, 100 MHz) δ 158.0, 139.2, 114.2, 49.0, 40.6, 34.1, 33.9, 32.0, 30.4, 29.7, 29.6, 29.5, 29.2, 29.0, 27.0, 25.7, 25.1 ppm.

1-Phenyl-3-(undec-10-en-1-yl)urea (**5.3**). Using the general procedure outlined above for synthesis of urea **5.1**, amine **4** (0.56g, 1.87 mmol) was transformed into urea **5.3** (0.31 g, 57%) as a white solid; IR 3294, 3331, 2923, 2849, 1625, 1594 cm^{-1}; ^1H NMR ($CDCl_3$, 400 MHz) δ 7.56 (br, 1H), 7.24-7.18 (m, 4H), 6.97 (t, J = 7.2 Hz, 1H), 5.81-5.71 (m, 1H), 4.99-4.90 (m, 2H), 3.12 (q, J = 6.8 Hz, 2H), 2.01 (m, 2H), 1.39-1.20 (m, 14H) ppm; ^{13}C NMR ($CDCl_3$, 100 MHz) δ 156.8, 156,7, 139.3, 139.1, 129.1, 123.0, 120. 3, 114.2, 40.3, 33.9, 32.0, 30.3, 29.7, 29.6. 29.5, 29.4, 29.2, 29.0, 27.0, ppm.

1-(4-Fluorophenyl)-3-(undec-10-en-1-yl)urea (**5.4**). Using the general procedure outlined above for synthesis of urea **5.1**, amine **4** (0.49 g, 1.63 mmol) was transformed into urea **5.4** (0.32 g, 63%) as a white solid; IR 3331, 3305, 2922, 2848, 1624, 1606, 1563 cm^{-1}; ^1H NMR ($CDCl_3$, 400 MHz) δ 7.26-7.23 (m, 2H), 7.01 (t, J =8.8 Hz, 2H), 6.09 (br, 1H), 5.84-5.77 (m, 1H), 5.01-4.91 (m, 2H), 4.51 (br, 1H), 3.23 (q, J = 6.4 Hz, 2H), 2.03 (q, J = 6.8 Hz, 2H), 1.54-1.48 (m, 2H), 1.36-1.27 (m, 12H) ppm; ^{13}C NMR ($CDCl_3$, 100 MHz) δ 160.5, 158.1, 157.5, 139.2 x 2, 133.4, 121.5, 115.9, 115.6, 114.3, 33.9, 29.5, 29.4, 29.1, 29.0, 28.1, 26.0 ppm.

Intermediate thioester formations (step *b*, Scheme S2)

***S*-(11-(3-(*tert*-Butyl)ureido)undecyl) ethanethioate**. A solution of urea **5.1** (0.51 g, 1.90 mmol) and azobis(isobutyronitrile) (0.047 g, 0.28 mmol) in dry THF (20 mL) was heated to reflux whereupon thioacetic acid (0.25 mL, 3.51 mmol) was added via syringe. The reaction mixture was stirred at reflux 8h and then concentrated by rotary evaporation. The residue was purified by SiO_2 column chromatography, eluting with a 15:85 mixture of EtOAc:hexane, to afford the title thioester (0.28 g, 43%) as a pale yellow liquid; IR 3368, 3315, 2964, 2922, 2849, 1683, 1633, 1281 cm^{-1}; ^1H NMR ($CDCl_3$, 400 MHz) δ 4.13 (br, NH), 3.09 (t, J = 6.8 Hz, 2H), 2.85 (t, J = 7.6 Hz, 2H), 2.32 (s, 3H), 1.59-1.55 (m, 2H), 1.48-1.44 (m, 2H), 1.39-1.25 (m, 23H), ppm; ^{13}C NMR ($CDCl_3$, 100 MHz) δ 196.3, 157.8, 50.3, 40.7, 30.8, 30.7, 30.4, 29.7, 29.5 x 2, 29.4, 29.2, 29.1, 28.9, 27.0 ppm.

S-(11-((3-Cyclohexylureido)oxy)undecyl) ethanethioate. Using the general procedure outlined above for thioester formation, urea **5.2** (0.49 g, 1.66 mmol) was transformed into the title thioester (0.26 g, 42%) as a pale white solid; IR 3320, 2921, 2850, 1697, 1622, 1573, 1465 cm^{-1}; ^{1}H NMR ($CDCl_3$, 400 MHz) δ 4.59 (br, 2H), 3.51-3.46 (m, 1H), 3.09 (t, J = 6.4 Hz, 2H), 2.84 (t, J = 7.2 Hz, 2H), 2.30 (s, 3H), 1.91-1.80 (m, 2H), 1.68-1.66 (m, 2H),1.55-1.44 (m, 5H), 1.30-1.23 (m, 16H), 1.16-1.06 (m, 3H) ppm, ; ^{13}C NMR ($CDCl_3$, 100 MHz) δ 196.2, 157.9, 49.1, 49.0, 40.6, 34.1, 30.7 (2), 30.4, 29.5, 29.4, 29.2, 29.1, 28.8, 27.0,25.7, 25.0 ppm.

S-(11-(3-Phenylureido)undecyl) ethanethioate. Using the general procedure outlined above for thioester formation, urea **5.3** (0.26 g, 0.90 mmol) was transformed into the title thioester (0.17 g, 52%) as a white solid; IR 3332, 3294, 2919, 2849, 1691, 1625, 1594 cm^{-1}; ^{1}H NMR ($CDCl_3$, 400 MHz) δ 7.31 (m, 4H), 7.09-7.05 (m, 1H), H), 6.51 (br, 1H), 3.22 (t, J = 6.8 Hz, 2H), 2.85 (t, J = 7.6 Hz, 2H,), 2.31 (s, 3H), 1.56-1.50 (m, 4H), 1.48-1.24 (m, 14H), ppm; ^{13}C NMR ($CDCl_3$, 100 MHz) δ 196.5, 156.3, 139.0, 129.2, 123.4, 120.6, 40.4, 30.8, 30.2, 29.6, 29.5, 29.4, 29.2, 29.1, 28.9, 27.0 ppm.

S-(11-(3-(4-Fluorophenyl)ureido)undecyl) ethanethioate. Using the general procedure outlined above for thioester formation, urea **5.4** (0.20 g, 0.65 mmol) was transformed into the title thioester (0.11 g, 44%) as a white solid; IR 3325, 2921, 2849, 1695, 1627, 1562, 1504, 1518, 1216 cm^{-1}; ^{1}H NMR ($CDCl_3$, 400 MHz) δ 7.25-7.22 (m, 2H), 7.02-6.97 (m, 2H), 6.40 (br, NH), 4.77 (br, NH), 3.22 (t, J = 6.8 Hz, 2H), 2.85 (t, J = 7.61`Hz, 2H), 2.34 (s, 3H), 1.56-1.47 (m, 4H), 1.32-1.24 (m, 14H), ppm; ^{13}C NMR ($CDCl_3$, 100 MHz) δ 196.6, 160.1, 158.0, 156.4, 134.9 x 2, 122.6 x 2, 115.9, 115.7, 40.5, 30.8, 30.7, 30.2, 29.6, 29.5 x 2, 29.4, 29.2, 29.1, 28.9, 27.0 ppm.

Intermediate thioester cleavages (step c, Scheme S2)

1-(*tert*-butyl)-3-(11-mercaptoundecyl)urea (6.1). To a solution of the thioester prepared above (0.175 g, 0.50 mmol) in EtOH (5 mL) was added 12 N HCl (0.4 mL, 4.06 mmol). The reaction mixture was heated to reflux and stirred. After 4 h, the reaction mixture was cooled and then concentrated by rotary evaporation. The residue was dissolved in water (10 mL). The resultant solution was cooled to 0 °C and the pH was adjusted to slightly alkaline using saturated $NaHCO_3$ solution. The aqueous layer then was extracted with CH_2Cl_2 (4 X 30 mL). The combined organic phase was washed with saturated NaCl (2 X 20 mL), dried ($MgSO_4$), filtered

and concentrated under reduced pressure to afford thiol-urea **6.1** (0.115 g, 77%) as a pale yellow liquid that was directly applied to gold nanoparticles; IR 3315, 2919, 2850, 1631, 1562, 1467, 1453 cm^{-1}; ^{1}H NMR ($CDCl_3$, 400 MHz) δ 4.12 (br s, 2H), 3.09 (t, J = 6.8 Hz, 2H), 2.51 (q, J = 7.2 Hz, 2H), 1.63-1.58 (m, 2H), 1.47-1.44 (m, 2H), 1.38-1.26 (m, 23H) ppm; ^{13}C NMR ($CDCl_3$, 100 MHz) δ ppm 157.8, 50.3, 40.4, 34.1, 30.4, 30.2, 29.7, 29.6, 29.4, 29.1, 28.4, 27.0, 24.7 ppm.

1-Cyclohexyl-3-(11-mercaptoundecyl)urea (**6.2**). Using the general procedure outlined above for synthesis of thiol-urea **6.1**, the corresponding intermediate thioester (0.191g, 0.57 mmol) was transformed into thiol-urea **6.2** (0.15 g, 88%) as a white solid that was directly applied to gold nanoparticles; IR 3315, 2921, 2850, 1620, 1573, 1465, 1242 cm^{-1}; ^{1}H NMR ($CDCl_3$, 400 MHz) δ 4.16 (br s, 2H), 3.49 (br, 1H), 3,13 (t, J = 7.2 Hz, 2H), 2.52 (q, J = 7.6 Hz, 2H), 1.94-1.92 (m, 2H), 1.71-1.68 (m, 2H), 1.63-1.60 (m, 3H), 1.48-1.46 (m, 2H), 1.36-1.26 (m 16H), 1.19-1.1.08 (m, 3H) ppm; ^{13}C NMR ($CDCl_3$, 100 MHz) δ 158.0, 49.0, 48.9, 40.6, 34.1, 30.4, 29.6, 29.5, 29.1, 28.4, 27.0, 25.7, 25.1, 24.7 ppm.

1-(11-Mercaptoundecyl)-3-phenylurea (**6.3**). Using the general procedure outlined above for synthesis of thiol-urea **6.1**, the corresponding intermediate thioester (0.1 g, 0.27 mmol) was transformed into thiol-urea **6.3** (0.08 g, 89%) as a white solid that was directly applied to gold nanoparticles; IR 3331, 3294, 2921, 2849, 1625, 1594, 1558 cm^{-1}; ^{1}H NMR ($CDCl_3$, 400 MHz) δ 7.34-7.28 (m, 4H), 7.10 (d, J = 7.6 Hz, 2H), 3.24 (t, J =6.8 Hz, 2H), 2.51 (q, J = 7.2 Hz, 2H), 1.63-1.58 (m, 2H), 152-1.49 (m, 2H), 1.38-1.26 (m, 14H) ppm; ^{13}C NMR ($CDCl_3$, 100 MHz) δ 156.7, 138.9, 129.2, 123.5, 123.4, 120.2, 40.4, 34.1, 30.2, 29.6, 29.4, 29.1, 28.4, 27.0,24.7 ppm.

1-(4-Fluorophenyl)-3-(11-mercaptoundecyl)urea (**6.4**). Using the general procedure outlined above for synthesis of thiol-urea **6.1**, the corresponding intermediate thioester (0.13 g, 0.34 mmol) was transformed into thiol urea **6.4** (0.085 g, 74%) as a white solid that was directly applied to gold nanoparticles; IR 3326, 3297, 2921, 2847, 1623, 1603, 1561, 1518 cm^{-1}; ^{1}H NMR ($CDCl_3$, 400 MHz) δ 7.25-7.22 (m, 2H),), 7.01-6.97 (m, 2H), 6.44 (br, s, NH), 3.21 (t, J = 6.8 Hz, 2H), 2.51 (q, J = 7.2 Hz, 2H), 1.63-1.58 (m, 4H), 1.50-1.46 (m, 2H), 1.46-1.25 (m, 14H) ppm; ^{13}C NMR ($CDCl_3$, 100 MHz) δ 160.5, 158.1, 156.7, 134.1, 122.9, 122.8, 115.9, 115.7, 40.5, 34.1, 30.3, 29.6, 29.4, 29.1, 28.4, 27.0, 24.7 ppm.

Series III synthesis procedures

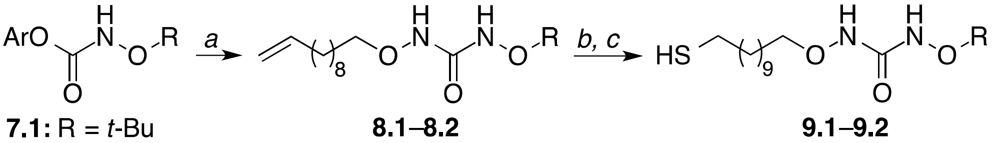

7.1: R = *t*-Bu
7.2: R = C_6H_{11}

Scheme S3. Synthesis of *N,N'*-dialkoxyurea thiols **9.1-9.2** (Series III). Reagents and conditions: *a*. $H_2C{=}CH(CH_2)_9ONH_2$ (fr. **1**), Et_3N, CH_2Cl_2, 0 °C to rt; *b*. $CH_3C(O)SH$, cat. AIBN, THF, reflux; *c*. HCl, EtOH, reflux.

General procedure for reagent activation with 4-nitrophenyl chloroformate

4-Nitrophenyl *tert*-butoxycarbamate (**7.1**). *O*-(*tert*-butyl)hydroxylamine hydrochloride (1.39 g, 11.11 mmol) was dissolved in CH_2Cl_2 (100 mL) followed by the addition of pyridine (1.2 mL, 13.89 mmol) under an argon atmosphere. The reaction flask was cooled to 0 °C and 4-nitrophenyl chloroformate (2.24 g, 11.11 mmol) was added. Then the reaction mixture was heated at reflux for 24 h. Upon cooling, the homogeneous organic solution was washed with 1 M $NaHCO_3$ (3 x 60 mL) and H_2O (2 x 40 mL). The organic fraction was dried with $MgSO_4$, filtered, and concentrated. The residue was purified by SiO_2 column chromatography, eluting with a 20:80 mixture of EtOAc:hexane, to afford **7.1** (1.96 g, 69%) as a white solid; IR 3281, 3073, 2984, 1737, 1615, 1520 cm^{-1}; ^1H NMR ($CDCl_3$, 400 MHz) δ 8.26 (d, J = 9.2 H, 2H), 7.34 (d, J = 8.8 Hz, 2H), 1.32 (s, 9H) ppm; ^{13}C NMR ($CDCl_3$, 100 MHz) δ 155.4, 154.9, 145.2, 125.3 (2), 122.0, 121.9, 86.2, 26.2 ppm.

4-Nitrophenyl (cyclohexyloxy)carbamate (**7.2**). Using the general procedure outlined above, *O*-(cyclohexyl)hydroxylamine hydrochloride (1.13 g, 9.80 mmol) was transformed into **7.2** (1.39 g, 50%) as a white solid; IR 3284, 2933, 2857, 1733, 1539, 1472 cm^{-1}; ^1H NMR ($CDCl_3$, 400 MHz) δ 8.26 (d, J = 8.0 Hz, 2H), 7.60 (br, 1H), 7.35 (d, J = 8.0 Hz, 2H), 3.89 (br,1H), 1.98 (br, 2H), 1.79 (br, 2H), 1.58-45 (m, 3H), 1.34-1.28 (m, 3H) ppm; ^{13}C NMR ($CDCl_3$, 100 MHz) δ 155.4, 154.2, 145.3, 125.4, 122.1, 121.9, 84.3, 84.2, 30.6, 25.6, 23.8 ppm.

Intermediate urea formations (step a, Scheme S3)

1-(*tert*-Butyloxy)-3-(undec-10-en-1-yloxy)urea (**8.1**). To a stirred solution of the crude aminooxy product derived from hydrazinolysis of phthalimide **1** (0.45 g, 2.43 mmol) in dry

CH_2Cl_2 (20 mL) at rt under argon was added dry Et_3N (0.68 mL, 4.86 mmol) via syringe. The reaction mixture was cooled to 0 °C and 4-nitrophenyl *tert*-butoxycarbamate (**7.1**) (1.85 g, 7.29 mmol) was added. The reaction mixture was heated at reflux 8h, whereupon the reaction mixture was allowed to cool to room temperature and quenched by addition of water (50 mL) and then extracted with CH_2Cl_2 (3 X 100 mL). The combined organic phase was dried over anhydrous $MgSO_4$ and concentrated under reduced pressure. The crude product was purified by column chromatography using gradient elution (4:1 to 3:2, hexane:ethyl acetate) to afford dialkoxyurea **8.1** (0.58 g, 76%) as a pale yellow liquid; IR 3216, 2925, 2854, 1688, 1466 cm^{-1}; ^{1}H NMR ($CDCl_3$, 400 MHz) δ 7.75 (br, NH), 7.09 (br, NH), 5.84-5.77 (m, 1H), 5.00-4.91 (m, 2H), 3.87 (t, J = 6.8 Hz, 2H), 2.03 (q, J = 6.4 Hz, 2H), 1.68-1.61 (m, 2H), 1.36-1.26 (m, 21H) ppm; ^{13}C NMR ($CDCl_3$, 100 MHz) δ 161.2, 139.3, 114.2, 81.5, 77.2, 33.8, 29.4, 29.1, 28.9, 28.0, 26.2, 25.9 ppm.

1-(Cyclohexyloxy)-3-(undec-10-en-1-yloxy)urea (**8.2**). Using the general procedure outlined above for synthesis of urea **8.1**, the crude aminooxy product derived from hydrazinolysis of phthalimide **1** (0.52 g, 1.64 mmol) was transformed into urea **8.2** (0.44 g, 83%) as a colorless liquid; IR 3221, 2926, 2855, 1687, 1466 cm^{-1}; ^{1}H NMR ($CDCl_3$, 400 MHz) δ 7.92 (s, NH), 7.74 (s, NH), 5.80-5.75 (m, 1H), 4.99-4.89 (m, 2H), 3.86 (t, J = 7.2 Hz, 2H), 3.73-3.71 (m, 1H), 2.04-1.95 (m, 4H), 1.76-1.74 (m, 2H), 1.64-1.53 (m, 3H), 1.39-1.19 (m, 15H) ppm; ^{13}C NMR ($CDCl_3$, 100 MHz) δ 160.7, 139.3, 139.2, 114.2, 83.7, 83.5, 77.2, 33.8, 30.7, 29.5, 29.4, 29.1, 29.0, 28.0, 25.9, 25.6, 23.9 ppm.

Intermediate thioester formations (step *b*, Scheme S3)

***S*-(11-((3-(*tert*-Butyl)ureido)oxy)undecyl) ethanethioate**. A solution of urea **8.1** (0.48 g, 1.59 mmol) and AIBN (0.04 g, 0.24 mmol) in dry THF (20 mL) was heated at reflux whereupon thioacetic acid (0.23 mL, 3.19 mmol) was added via syringe. The reaction mixture was stirred at reflux 8h and then cooled before concentrating by rotary evaporation. The residue was purified by SiO_2 column chromatography, eluting with a 15:85 mixture of EtOAc:hexane, to afford the corresponding thioester (0.45 g, 75%) as a colorless liquid; IR 3221, 2926, 2854, 1691 cm^{-1}; ^{1}H NMR ($CDCl_3$, 400 MHz) δ 7.71 (br, NH), 7.03 (br, NH), 3.87 (t, J = 6.0 Hz, 2H), 2.85 (t, J = 7.2 Hz, 2H), 2.31 (s, 3H), 1.66-1.63 (m, 2H), 1.57-1.53 (m, 2H), 1.33-1.26 (m, 23H) ppm; ^{13}C NMR ($CDCl_3$, 100 MHz) δ 196.0, 161.1, 81.3, 77.2, 30.6, 29.4. 29.1, 29.0, 28.8, 28.0, 26.2, 25.8 ppm.

***S*-(11-((3-(Cyclohexyloxy)ureido)oxy)undecyl) ethanethioate**. Using the general procedure outlined above for thioester formation, urea **8.2** (0.4 g, 1.22 mmol) was transformed into the title thioester (0.36 g, 73%) as a colorless liquid; IR 3215, 2924, 2853, 1685 cm^{-1}; ^{1}H NMR ($CDCl_3$, 400 MHz) δ 7.70 (br, NH), 7.49 (br, NH), 3.86 (t, J = 6.8 Hz, 2H), 3.76-3.71 (m, 1H), 2.85 (t, J = 7.6 Hz, 2H), 2.31 (s, 3H), 1.99-1.95 (m, 2H), 1.77-1.74 (m, 2H), 1.67-1.51 (m, 5H), 1.37-1.18 (m, 20H) ppm; ^{13}C NMR ($CDCl_3$, 100 MHz) δ 196.2, 160.6, 83.7, 83.6, 77.2, 30.7, 29.5, 29.2, 29.1, 28.9, 28.0, 25.9, 25.6, 23.9 ppm.

Intermediate thioester cleavages (step c, Scheme S3)

1-(*tert*-Butoxy)-3-((11-mercaptoundecyl)oxy)urea (**9.1**). To a solution of the thioester prepared above (0.40 g, 1.06 mmol) in EtOH (5 mL) was added 12 N HCl (0.83 mL, 8.5 mmol). The reaction mixture was heated at reflux 4h, whereupon the reaction mixture was cooled and then concentrated by rotary evaporation. The residue was dissolved in water (10 mL). The resultant solution was cooled to 0 °C and the pH was adjusted to slightly alkaline using saturated $NaHCO_3$ solution. The aqueous layer then was extracted with CH_2Cl_2 (4 X 30 mL). The combined organic phase was washed with saturated NaCl (2 X 20 mL), dried ($MgSO_4$), filtered and concentrated under reduced pressure to afford thiol-urea **9.1** (0.29 g, 81%) as a colorless gummy liquid that was directly applied to gold nanoparticles; ^{1}H NMR ($CDCl_3$, 400 MHz) δ 7.82 (br, NH), 7.19 (br, NH), 3.86 (t, J = 7.2 Hz, 2H), 2.51 (q, J = 7.6 Hz, 2H), 1.66-1.57 (m, 4H), 1.33-1.25 (m, 23H) ppm; ^{13}C NMR ($CDCl_3$, 100 MHz) δ 161.5, 81.3, 77.3, 34.1, 29.5, 29.4, 29.1, 28.4, 28.1, 26.3, 26.0, 25.9, 24.7 ppm.

1-(Cyclohexyloxy)-3-((11-mercaptoundecyl)oxy)urea (**9.2**). Using the general procedure outlined above for synthesis of thiol-urea **9.1**, the corresponding intermediate thioester (0.33g, 0.82 mmol) was transformed into thiol-urea **9.2** (0.185 g, 62%) as a colorless liquid. that was directly applied to gold nanoparticles; IR 3268, 3221, 2915, 2849, 1691, 1666, 1505 cm^{-1}; ^{1}H NMR ($CDCl_3$, 400 MHz) δ 7.77 (br, NH), 7.55 (br, NH), 3.87-3.84 (m, 2H), 3.73-3.72 (m, 1H), 2.51 (q, J = 7.6 Hz, 2H), 2.03-1.96 (m, 2H), 1.77-1.73 (m, 2H), 1.65-1.54 (m, 5H), 1.35-1.21 (m, 20H) ppm; ^{13}C NMR ($CDCl_3$, 100 MHz) δ 160.7, 83.6, 83.5, 77.2, 34.0, 30.7, 29.5, 29.4, 29.1, 28.4, 28.0, 25.8, 25.5, 24.7, 23.8 ppm.

III. ¹H and ¹³C NMR Spectra

¹H NMR spectrum (400 MHz, $CDCl_3$) of **1**

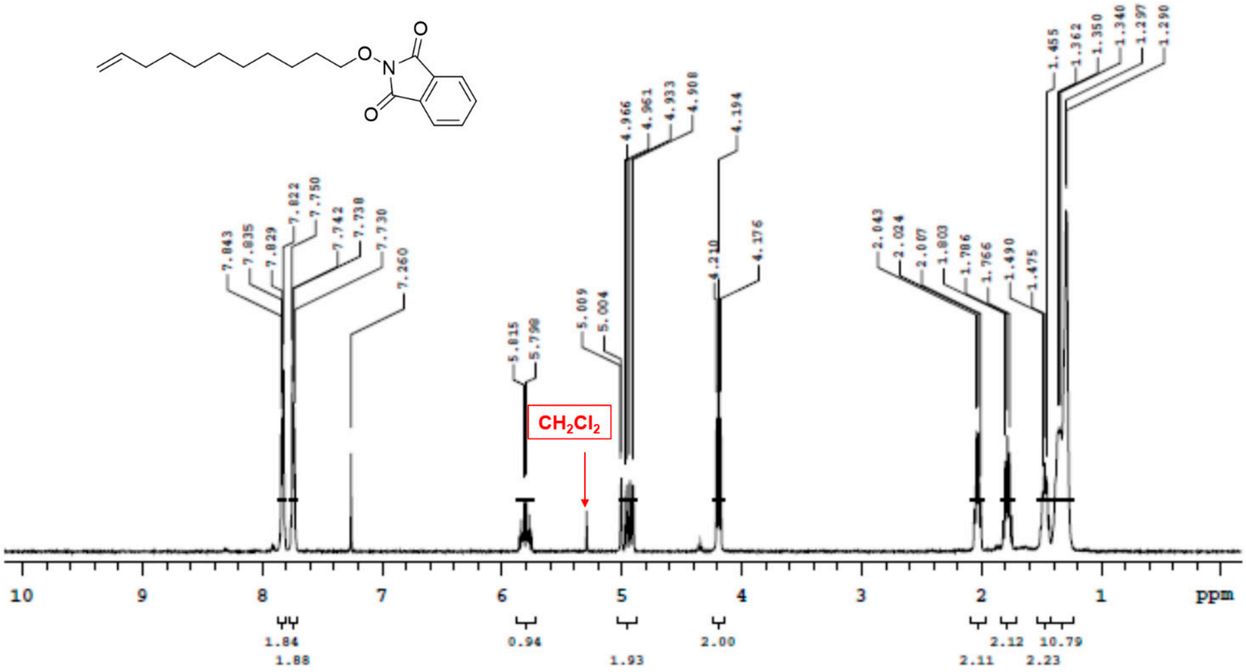

¹³C NMR spectrum (100 MHz, $CDCl_3$) of **1**

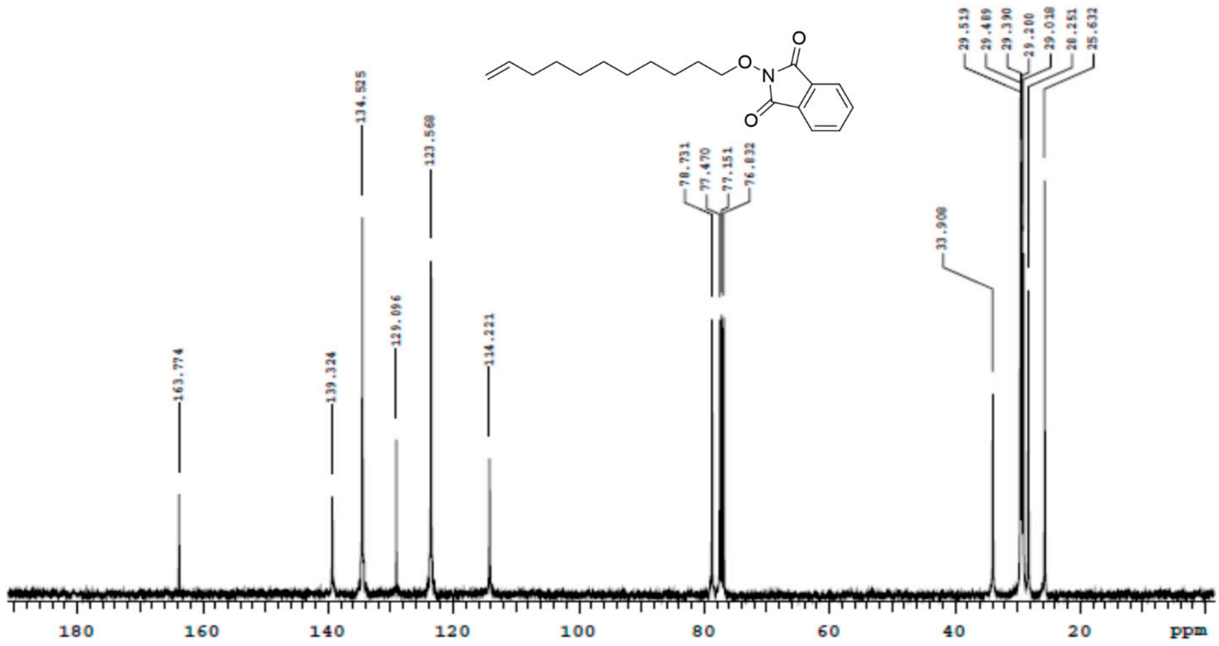

^{1}H NMR spectrum (400 MHz, $CDCl_3$) of thioester precursor to **3.1**

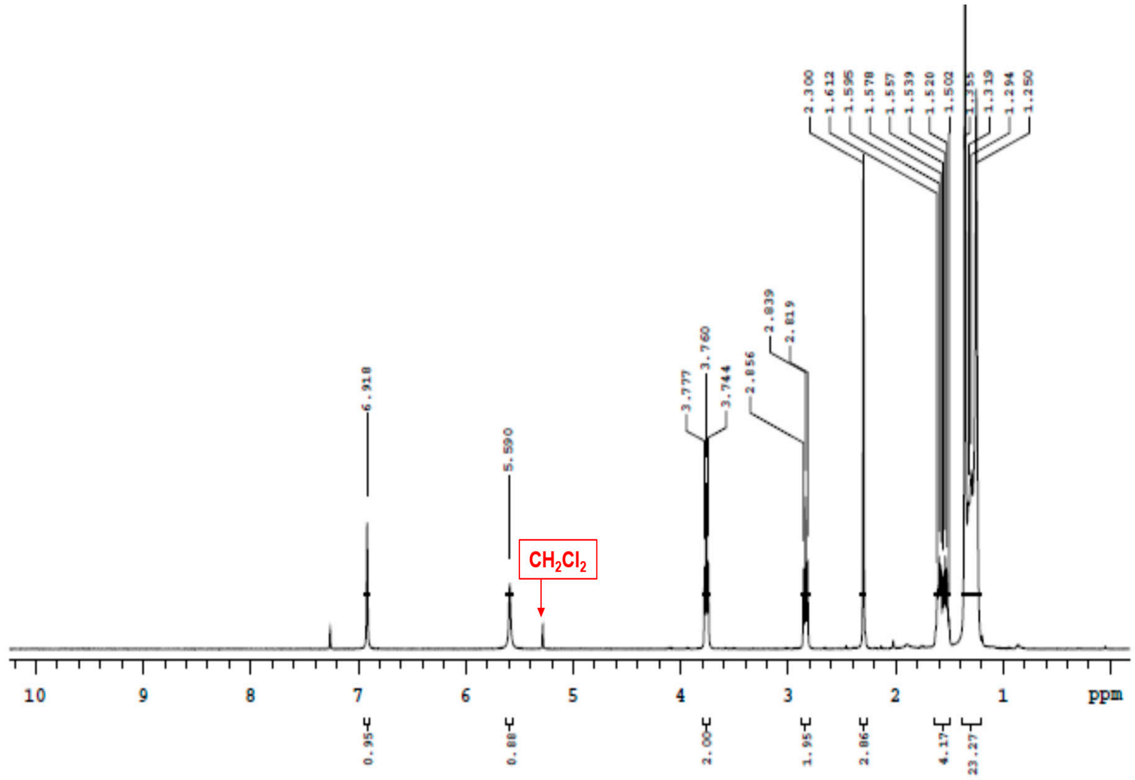

^{13}C NMR spectrum (400 MHz, $CDCl_3$) of thioester precursor to **3.1**

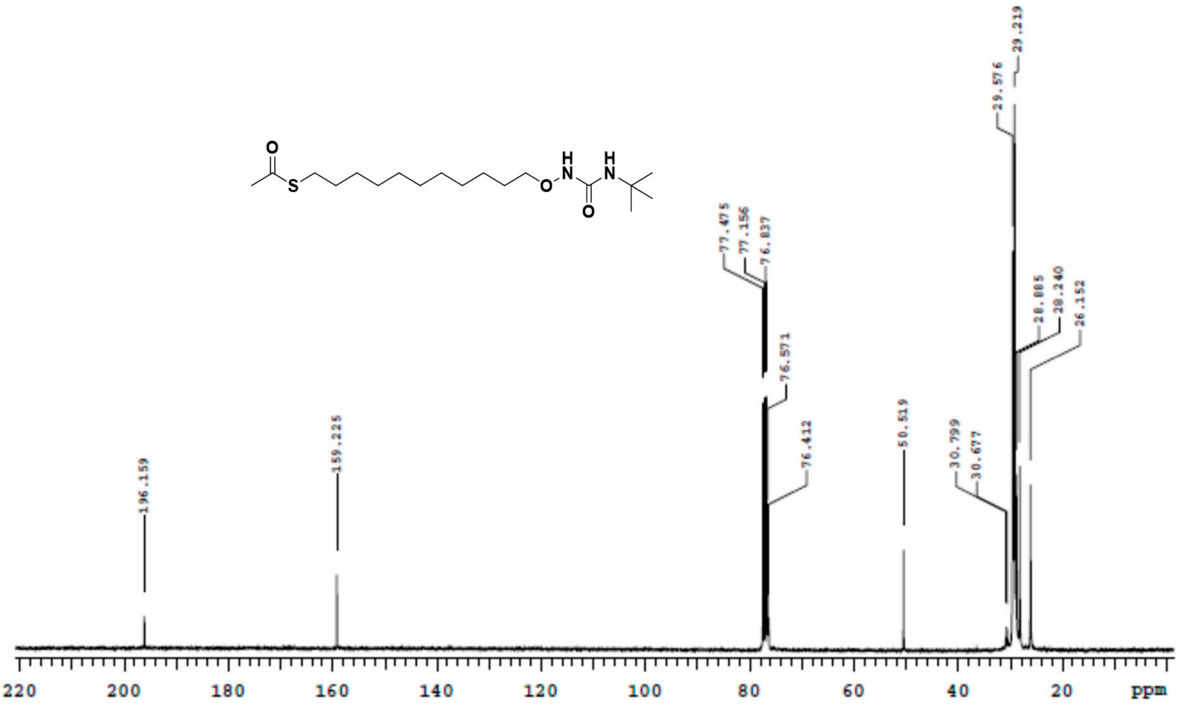

^{1}H NMR spectrum (400 MHz, $CDCl_3$) of **3.1**

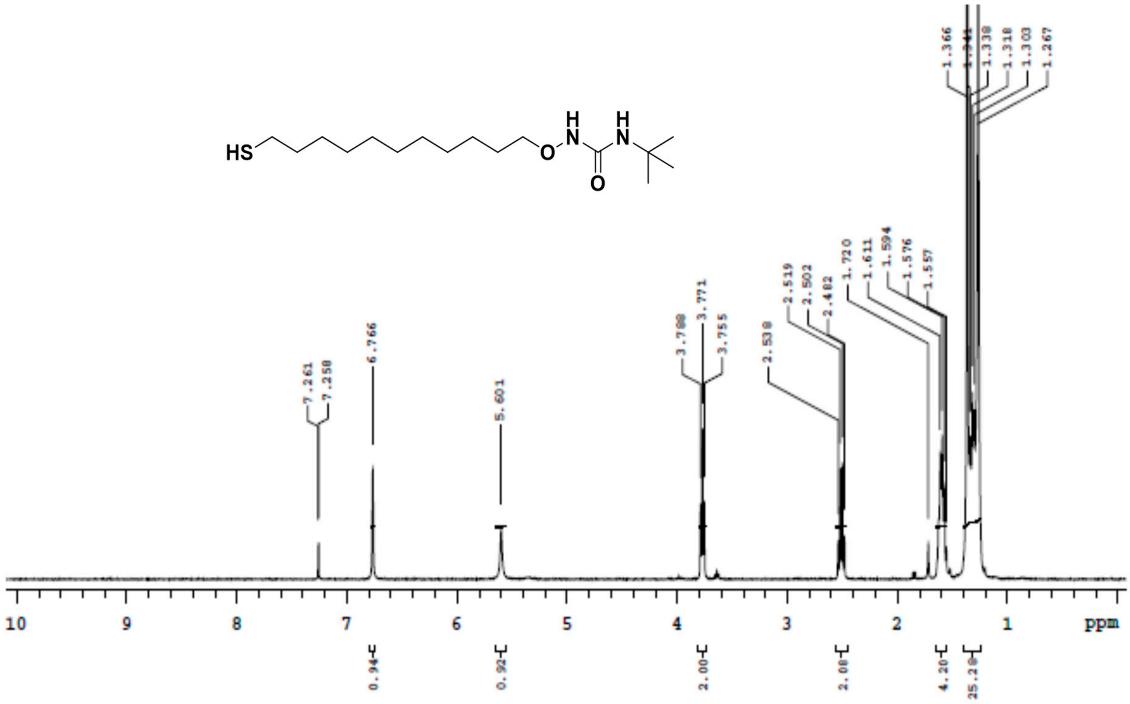

^{13}C NMR spectrum (400 MHz, $CDCl_3$) of **3.1**

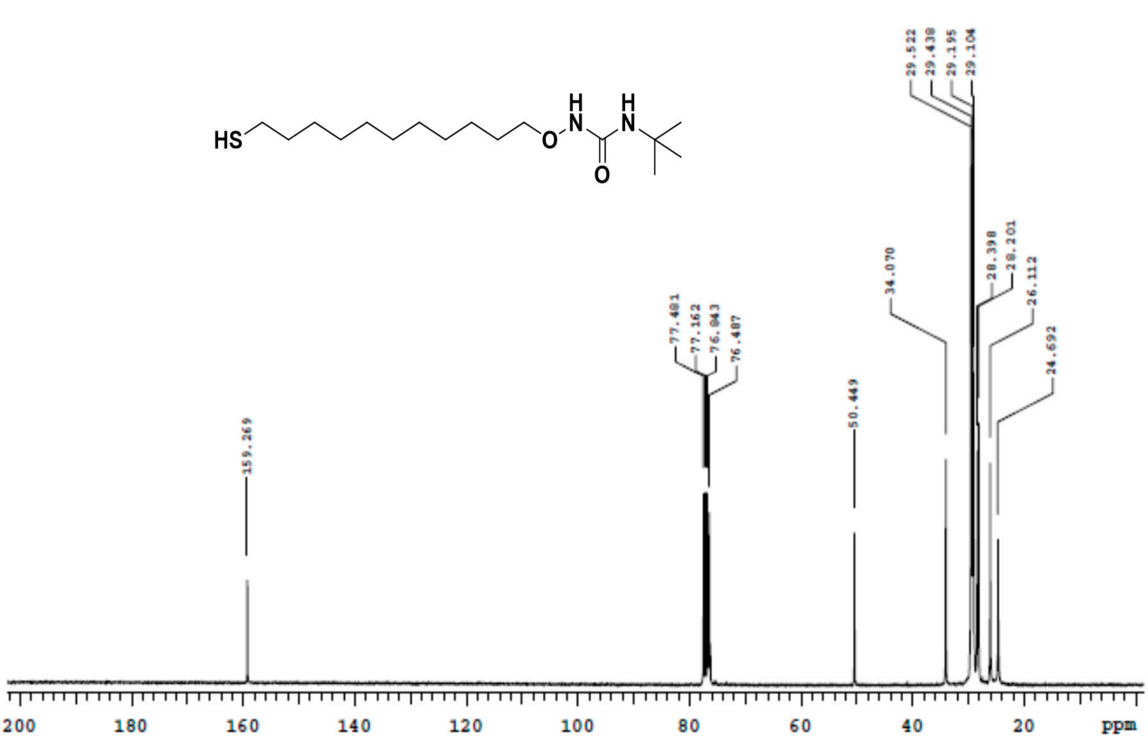

^{1}H NMR spectrum (400 MHz, $CDCl_3$) of thioester precursor to **3.2**

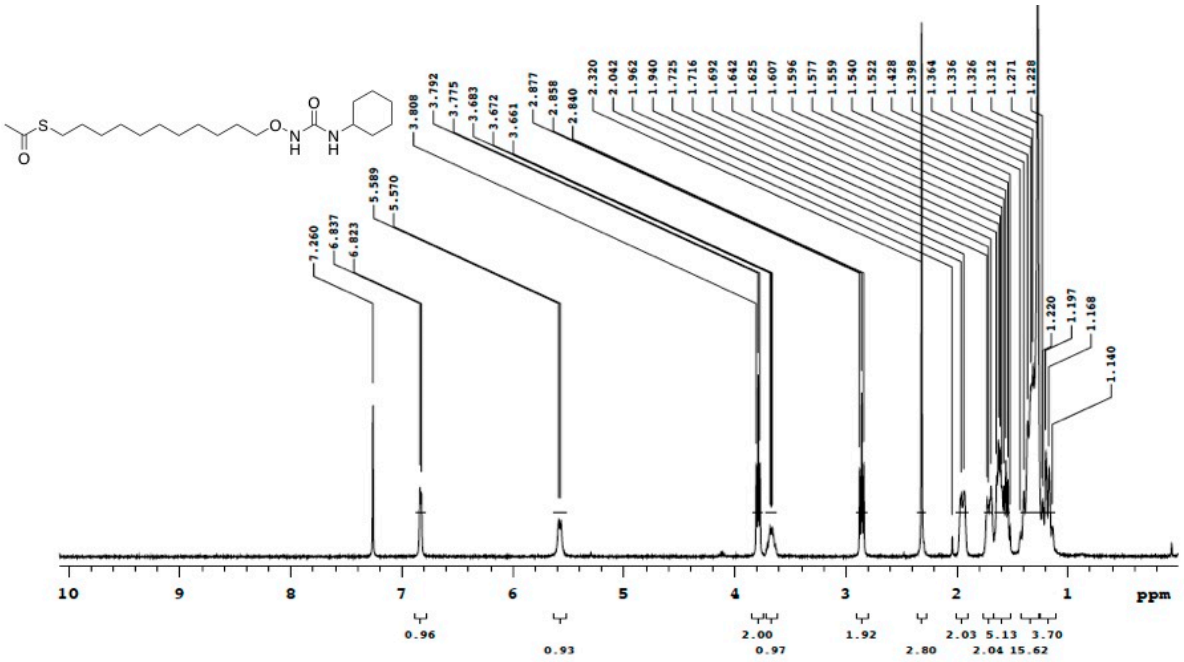

^{13}C NMR spectrum (100 MHz, $CDCl_3$) of thioester precursor to **3.2**

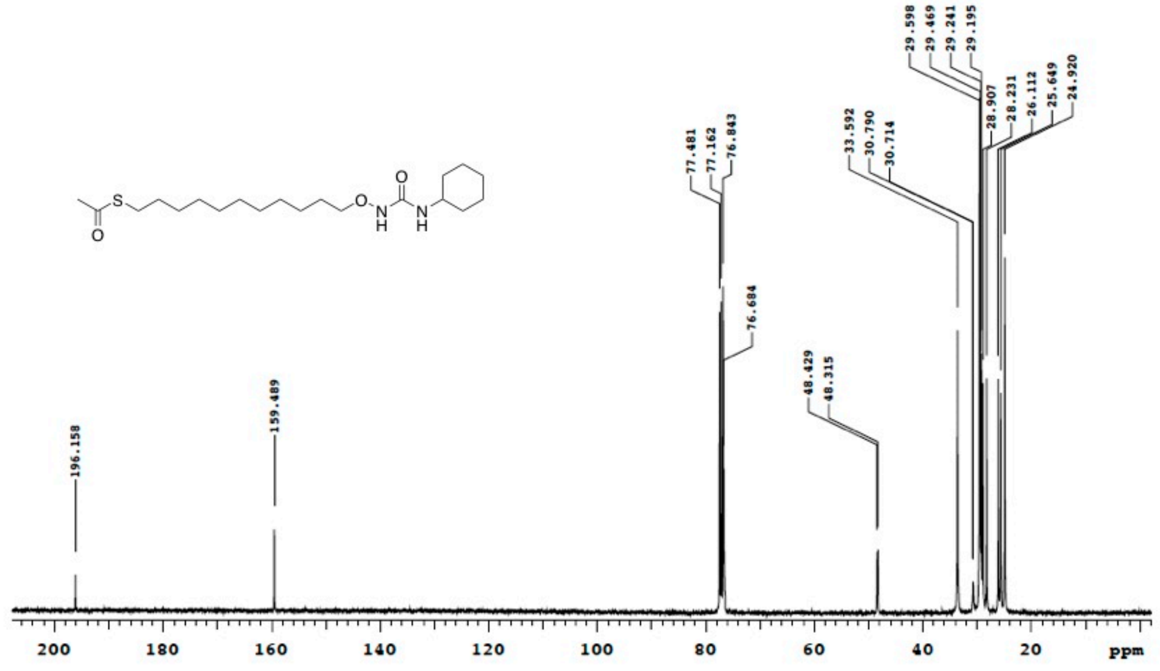

^{1}H NMR spectrum (400 MHz, $CDCl_3$) of **3.2**

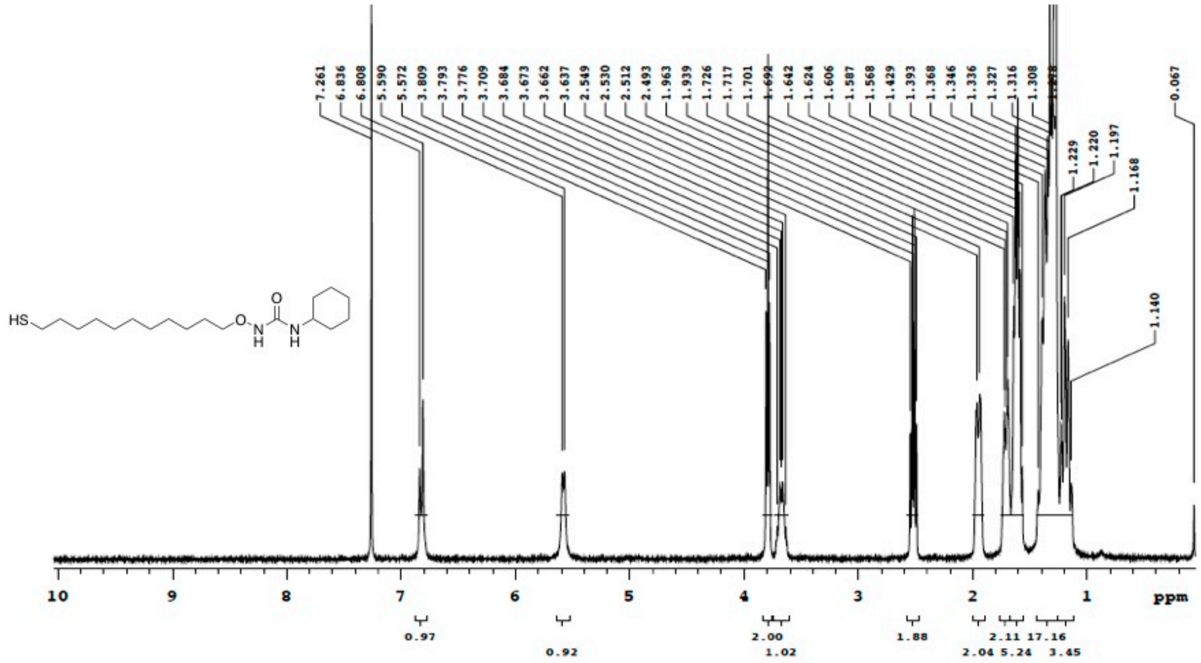

^{13}C NMR spectrum (100 MHz, $CDCl_3$) of **3.2**

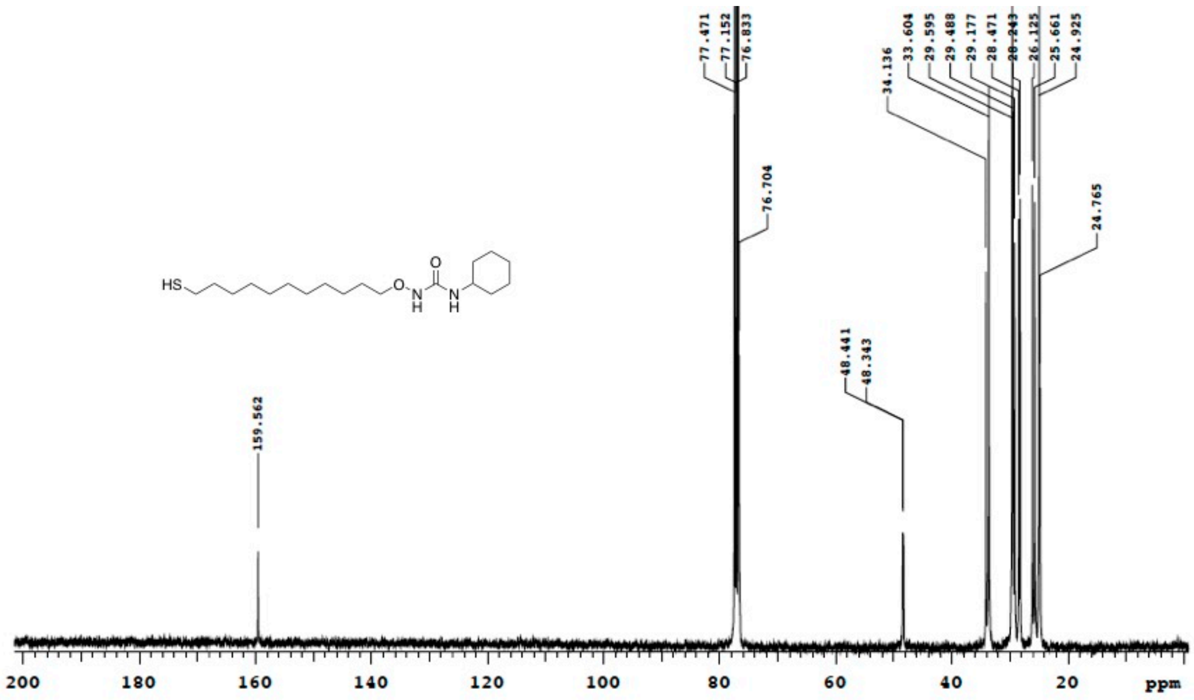

=

^{1}H NMR spectrum (400 MHz, $CDCl_3$) of thioester precursor to **3.3**

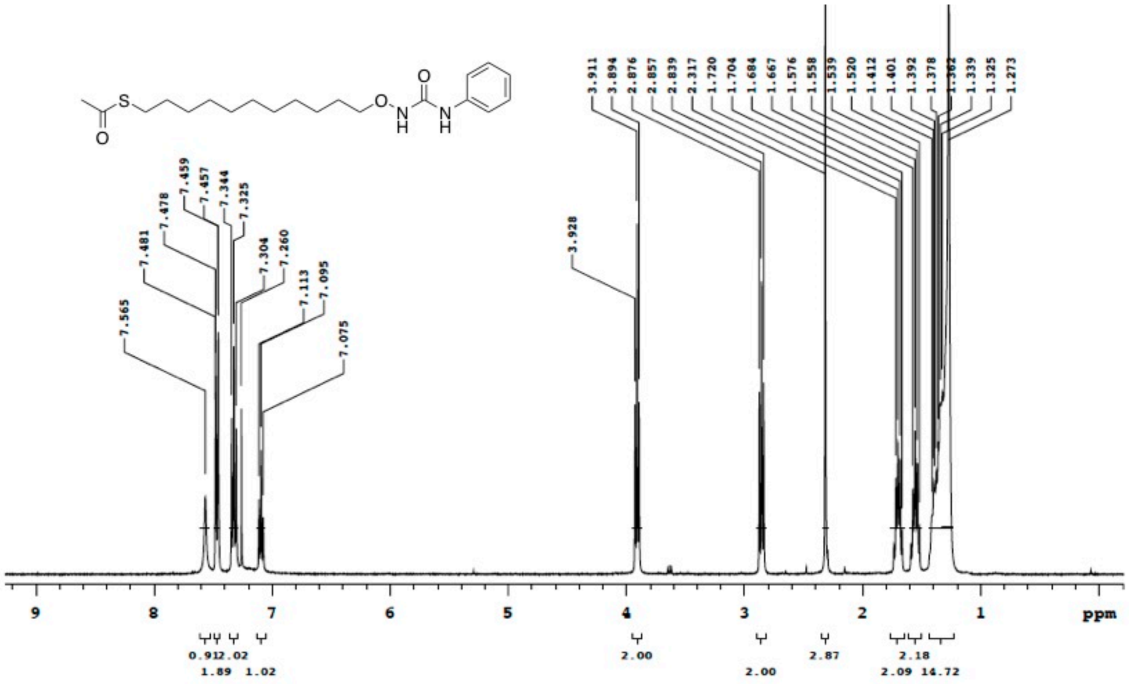

^{13}C NMR spectrum (100 MHz, $CDCl_3$) of thioester precursor to **3.3**

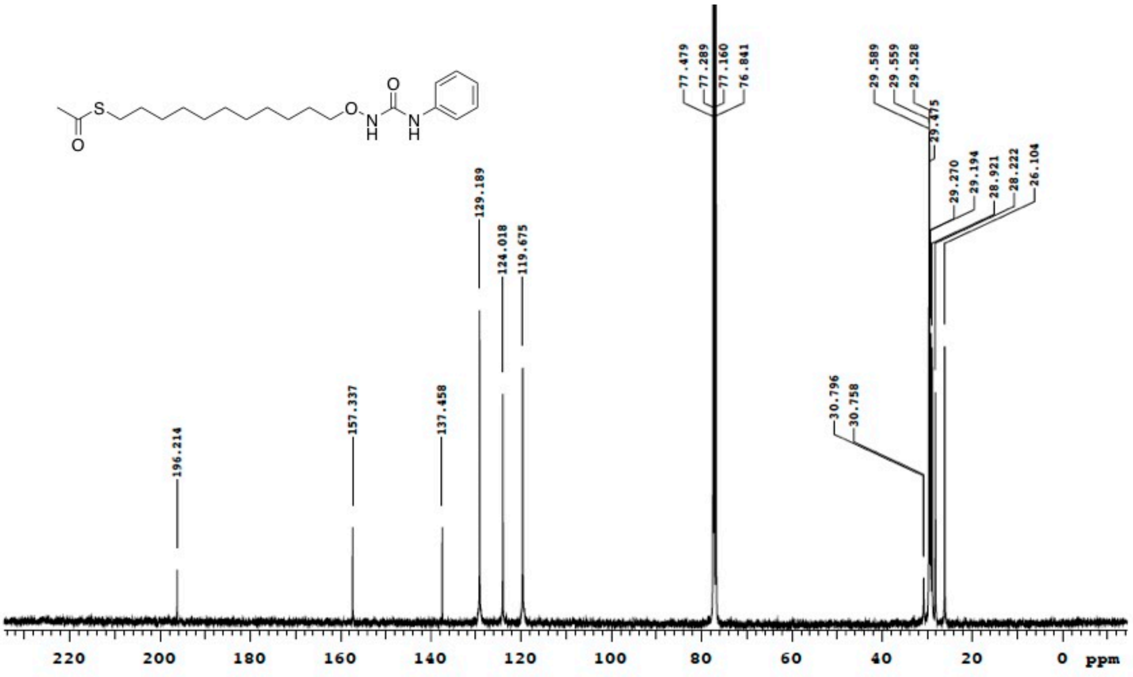

^{1}H NMR spectrum (400 MHz, $CDCl_3$) of **3.3**

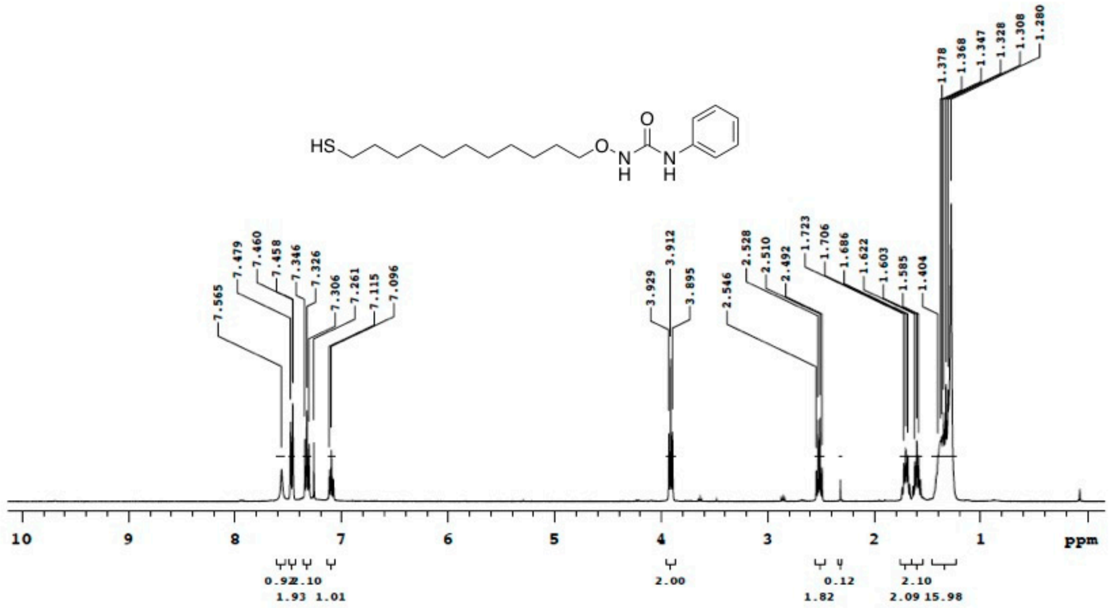

^{13}C NMR spectrum (100 MHz, $CDCl_3$) of **3.3**

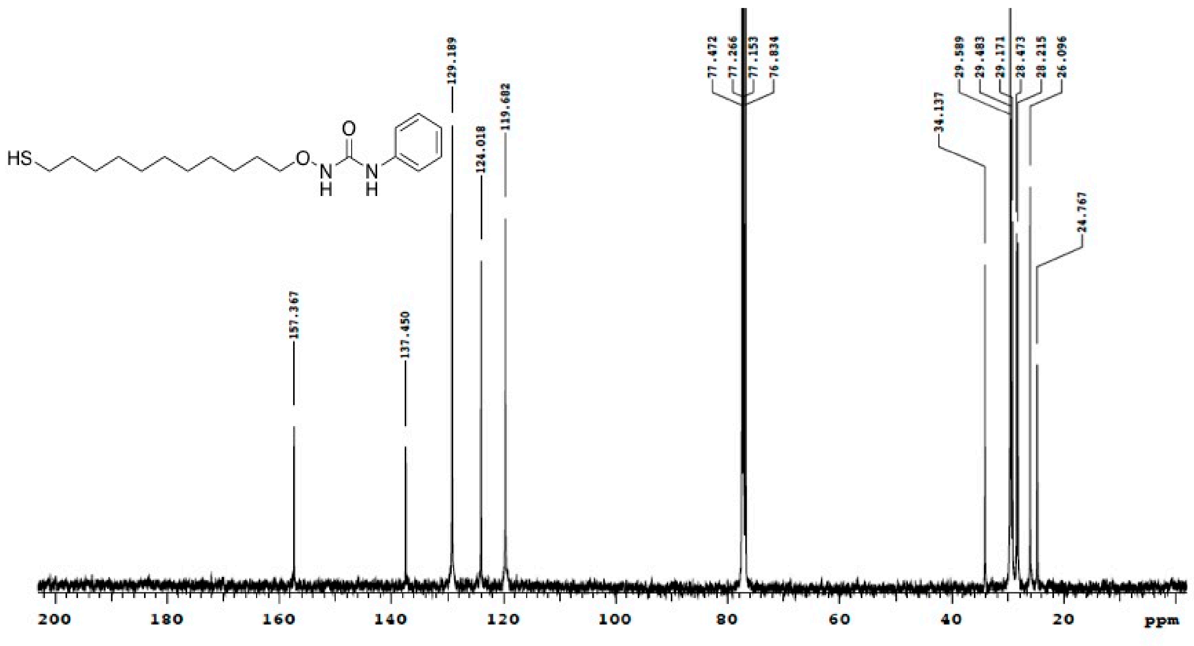

^{1}H NMR spectrum (400 MHz, $CDCl_3$) of thioester precursor to **3.4**

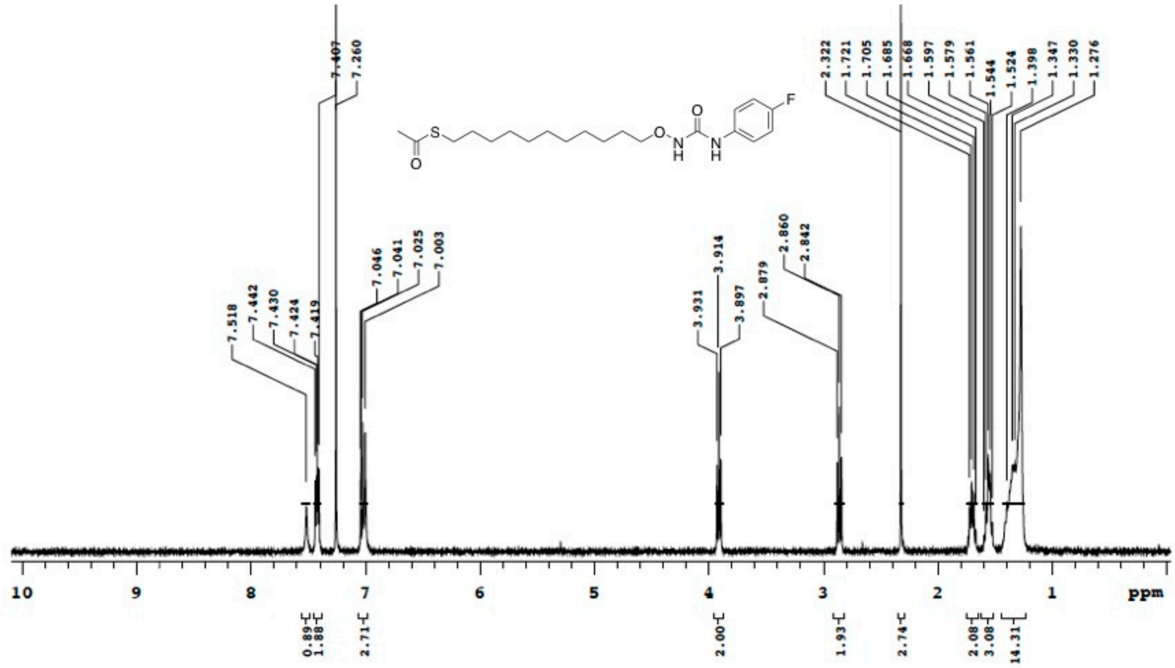

^{13}C NMR spectrum (100 MHz, $CDCl_3$) of thioester precursor to **3.4**

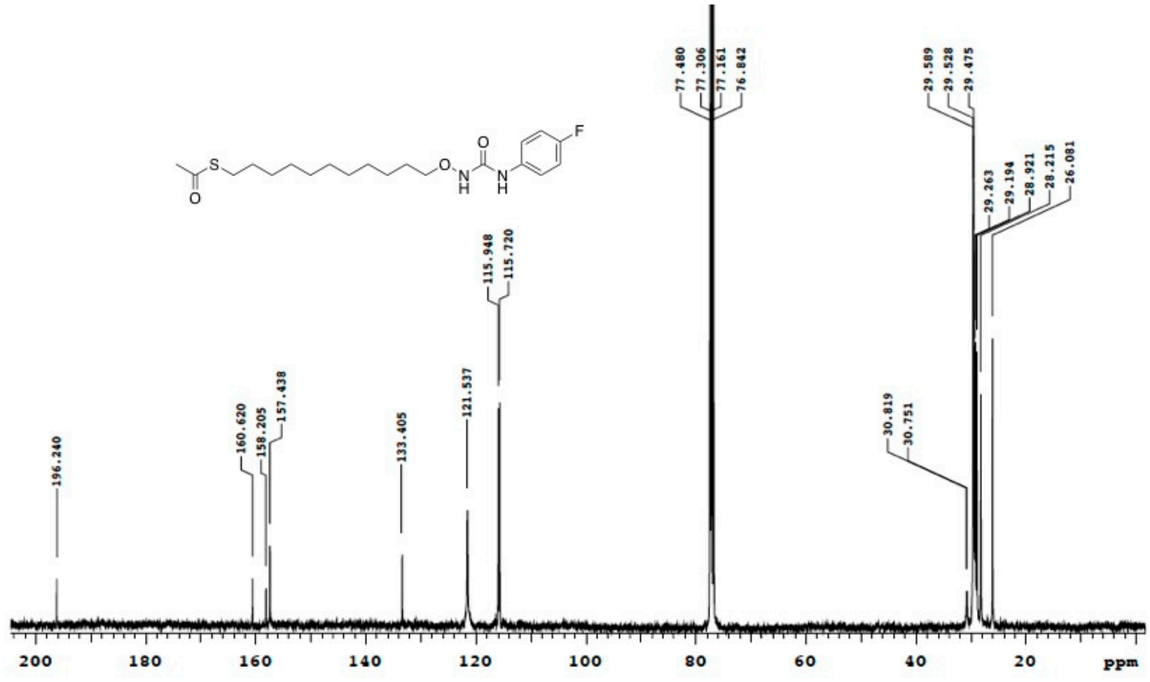

^{1}H NMR spectrum (400 MHz, $CDCl_3$) of **3.4**

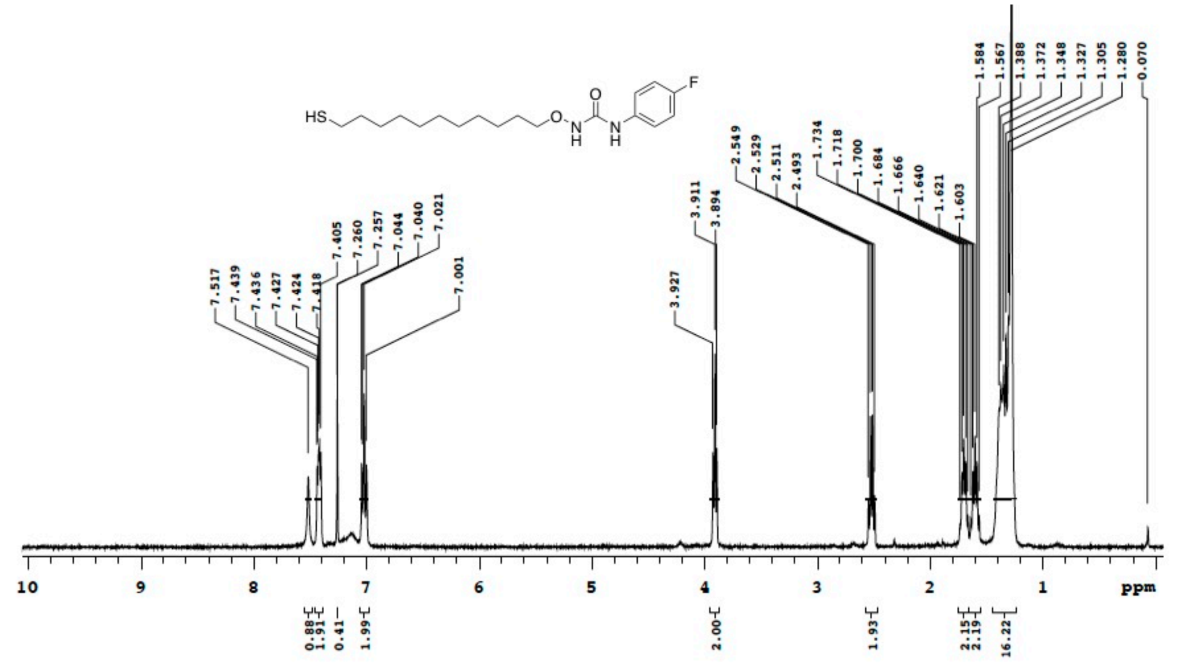

^{13}C NMR spectrum (100 MHz, $CDCl_3$) of **3.4**

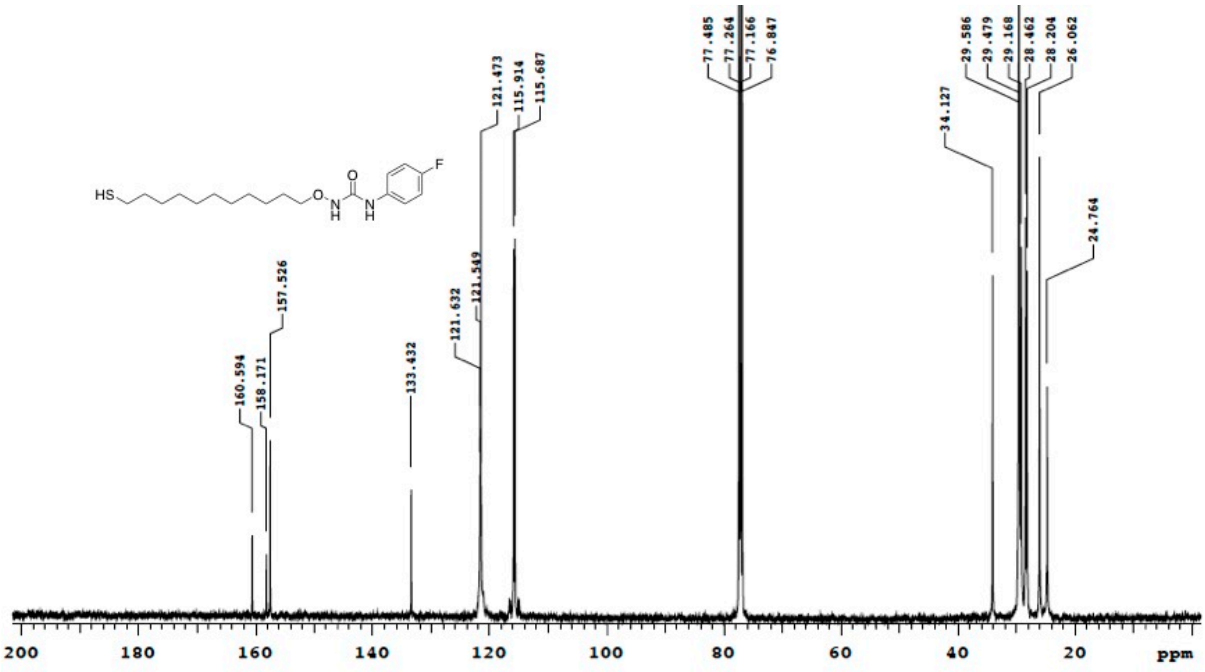

^{1}H NMR spectrum (400 MHz, $CDCl_3$) of thioester precursor to **6.1**

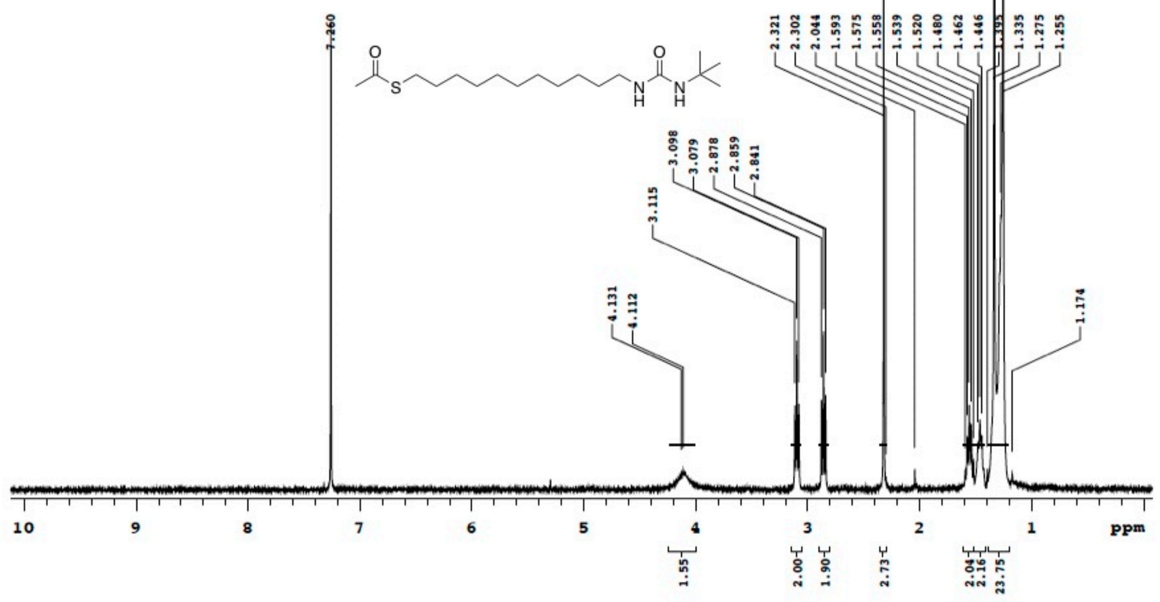

^{13}C NMR spectrum (100 MHz, $CDCl_3$) of thioester precursor to **6.1**

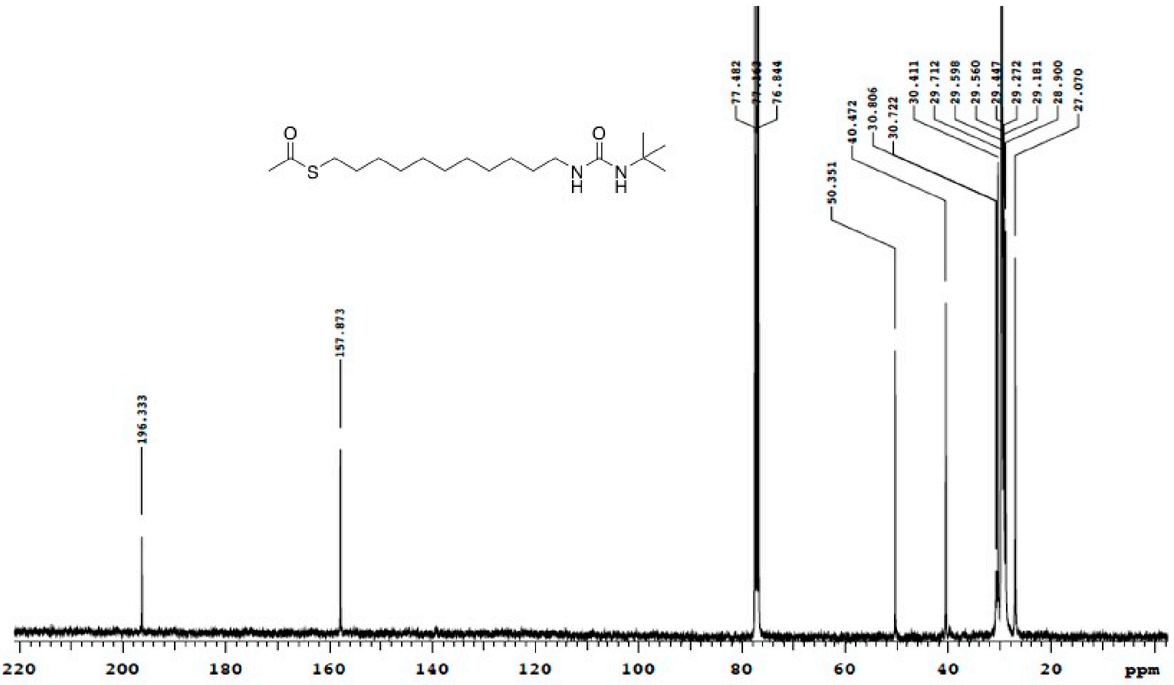

^{1}H NMR spectrum (400 MHz, $CDCl_3$) of **6.1**

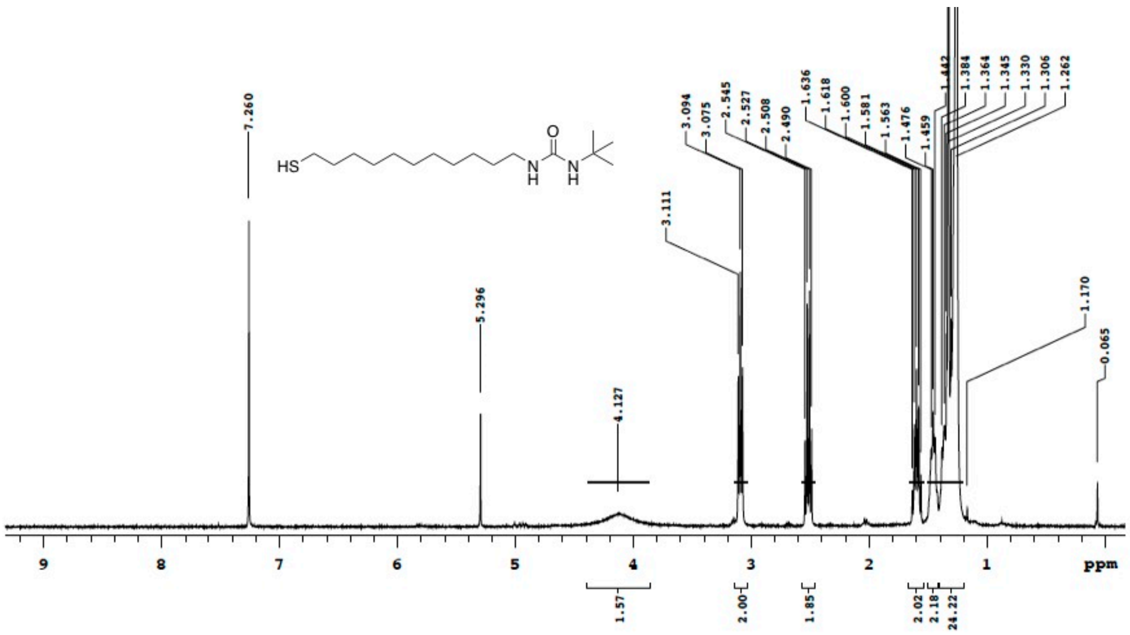

^{13}C NMR spectrum (100 MHz, $CDCl_3$) of **6.1**

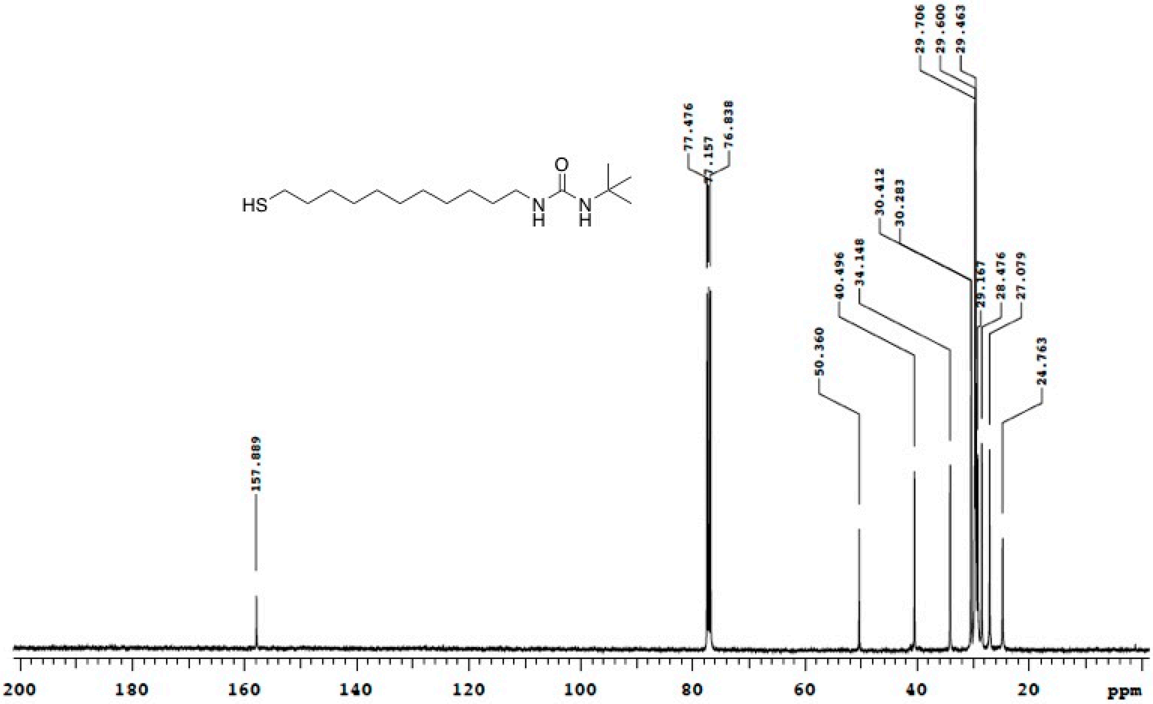

^{1}H NMR spectrum (400 MHz, $CDCl_3$) of thioester precursor to **6.2**

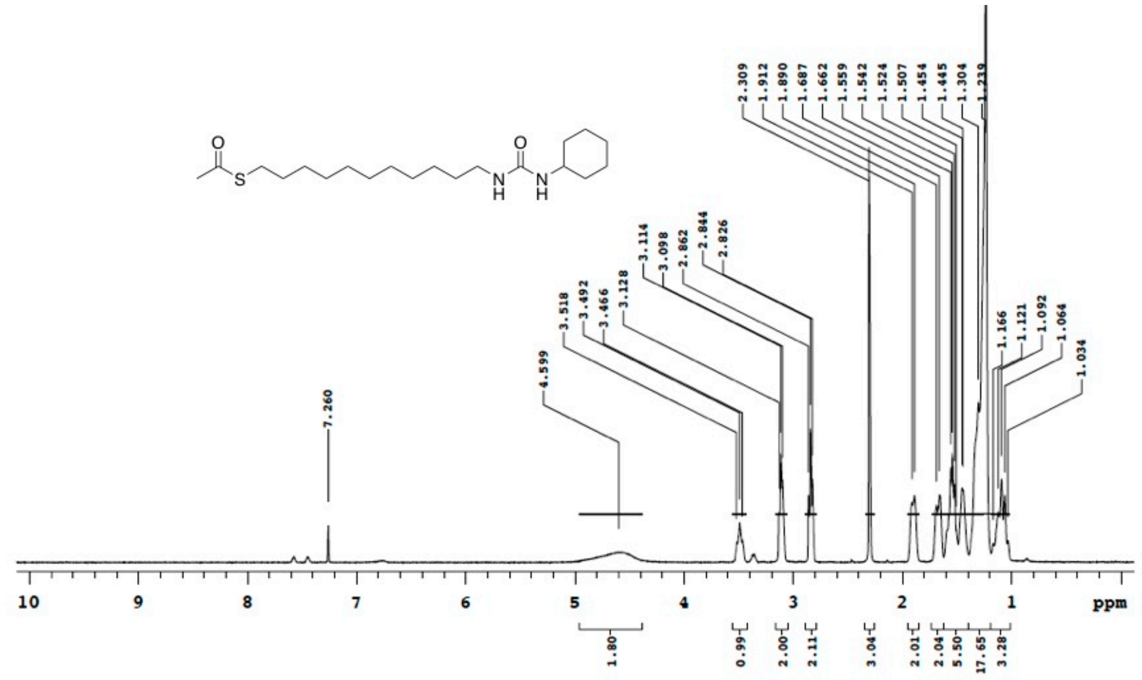

^{13}C NMR spectrum (100 MHz, $CDCl_3$) of thioester precursor to **6.2**

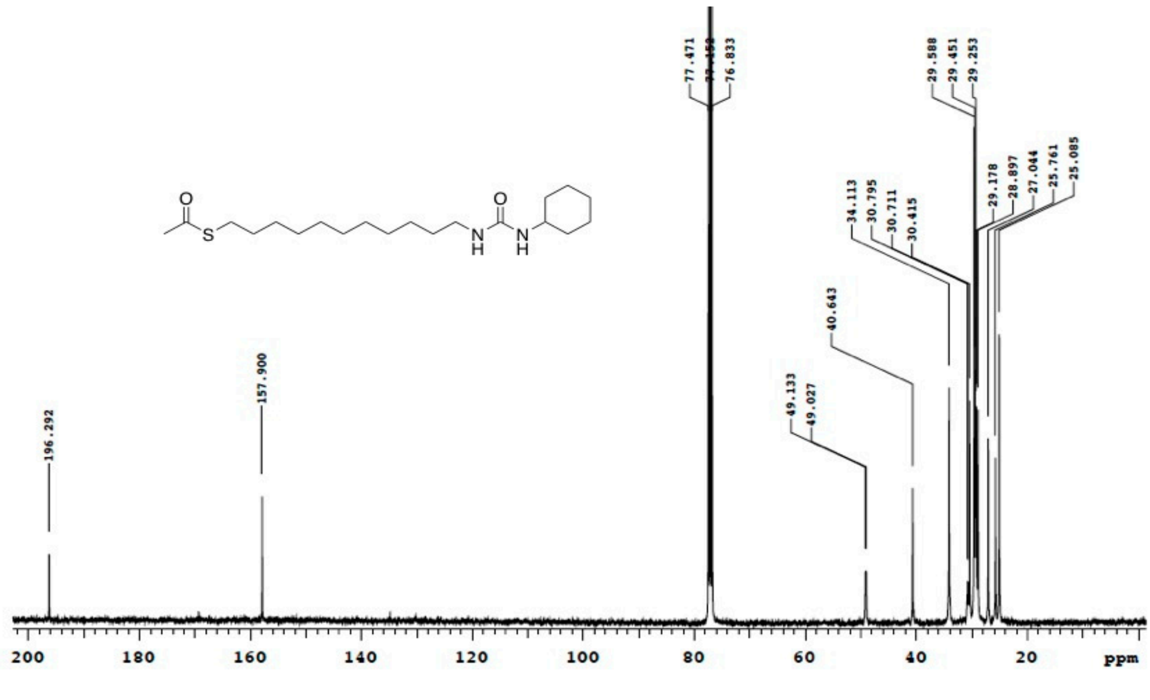

^{1}H NMR spectrum (400 MHz, $CDCl_3$) of **6.2**

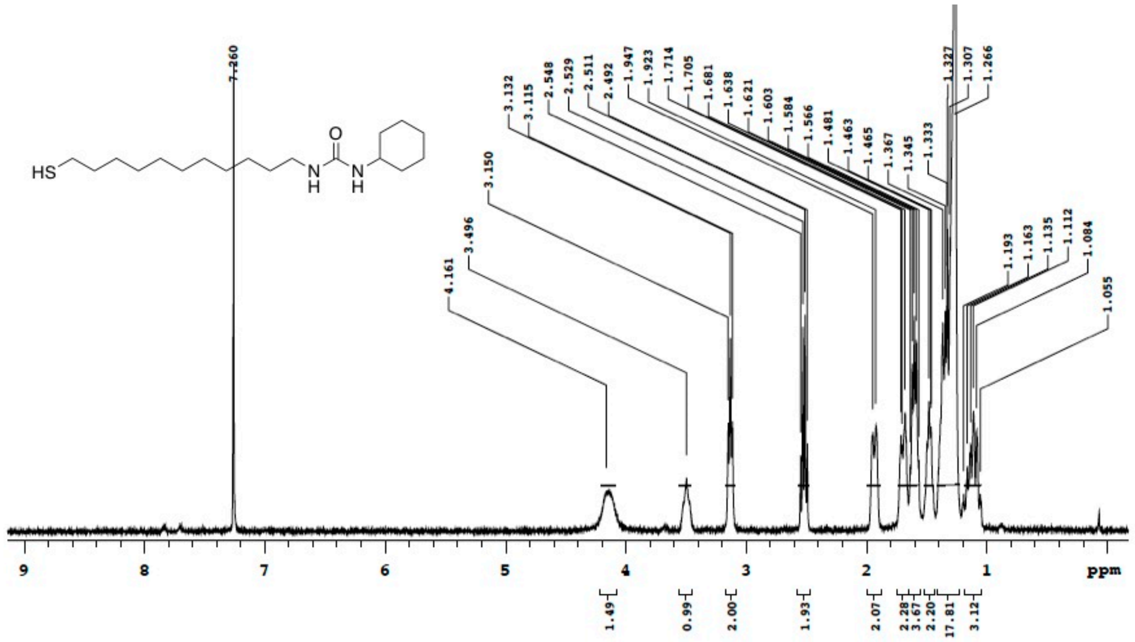

^{13}C NMR spectrum (100 MHz, $CDCl_3$) of **6.2**

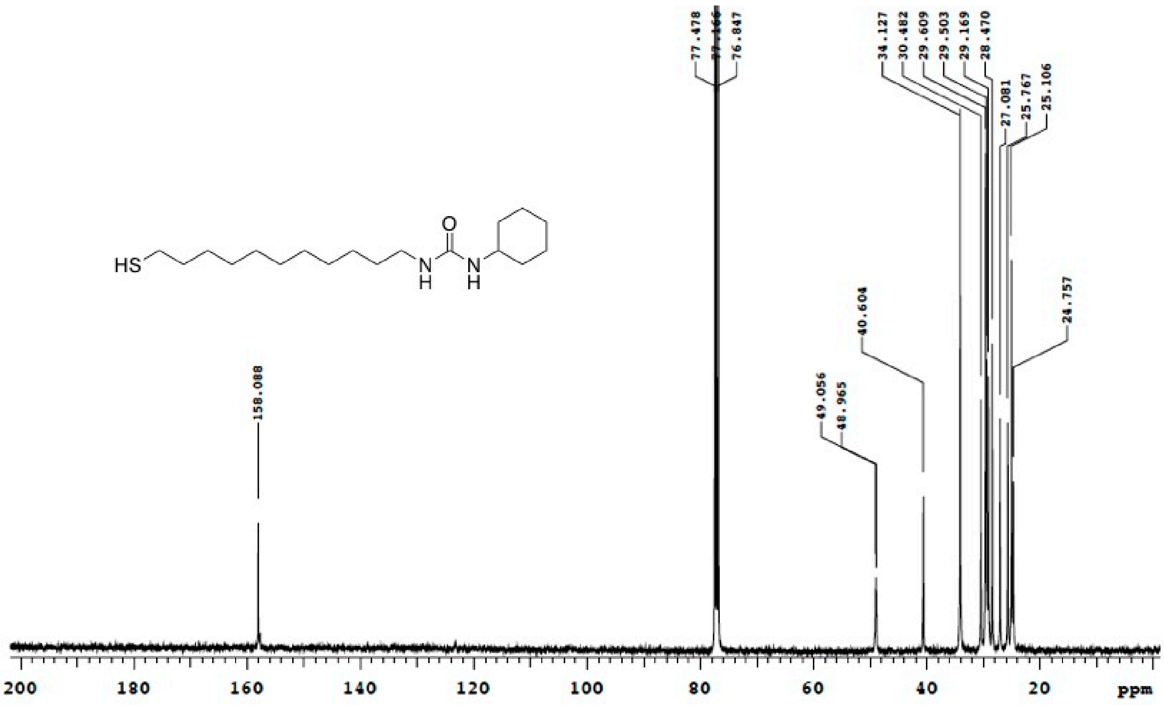

^{1}H NMR spectrum (400 MHz, $CDCl_3$) of thioester precursor to **6.3**

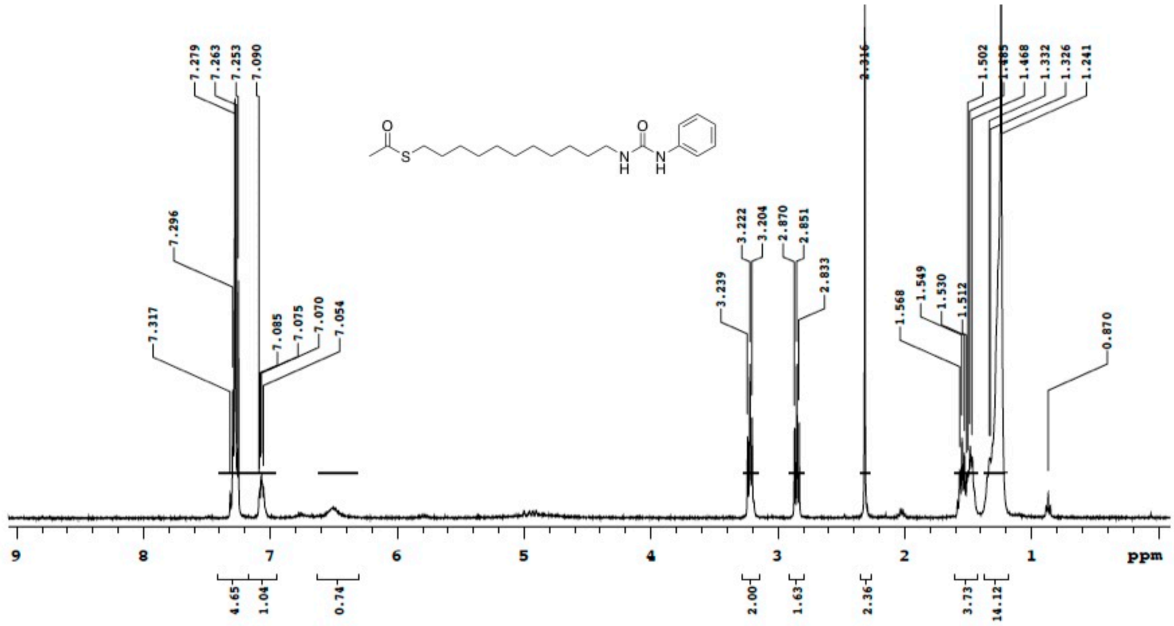

^{13}C NMR spectrum (100 MHz, $CDCl_3$) of thioester precursor to **6.3**

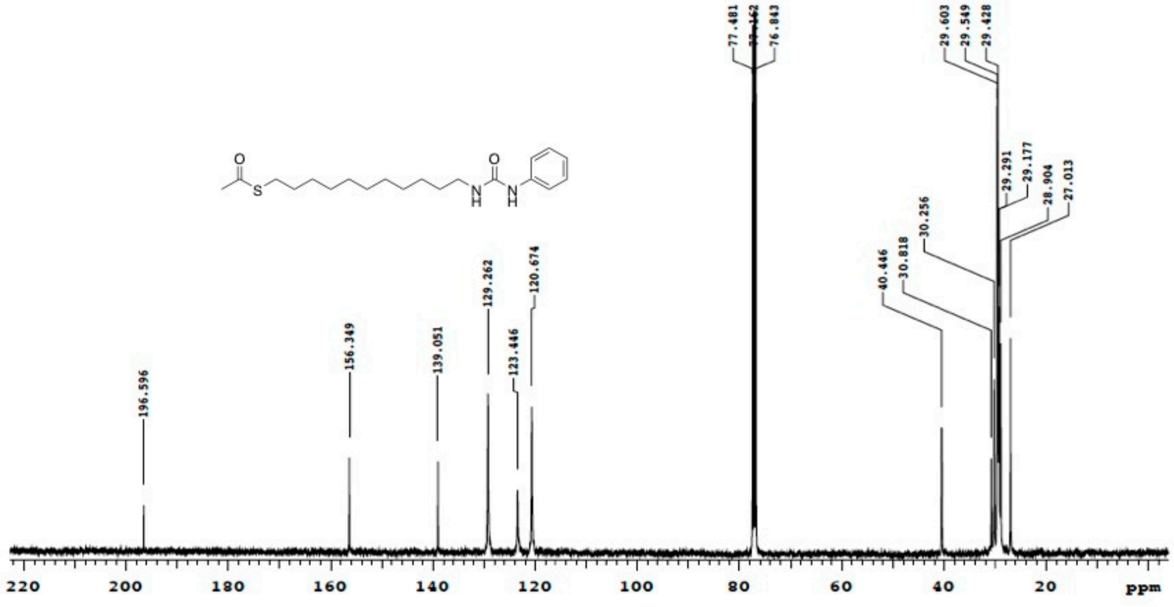

^{1}H NMR spectrum (400 MHz, $CDCl_3$) of **6.3**

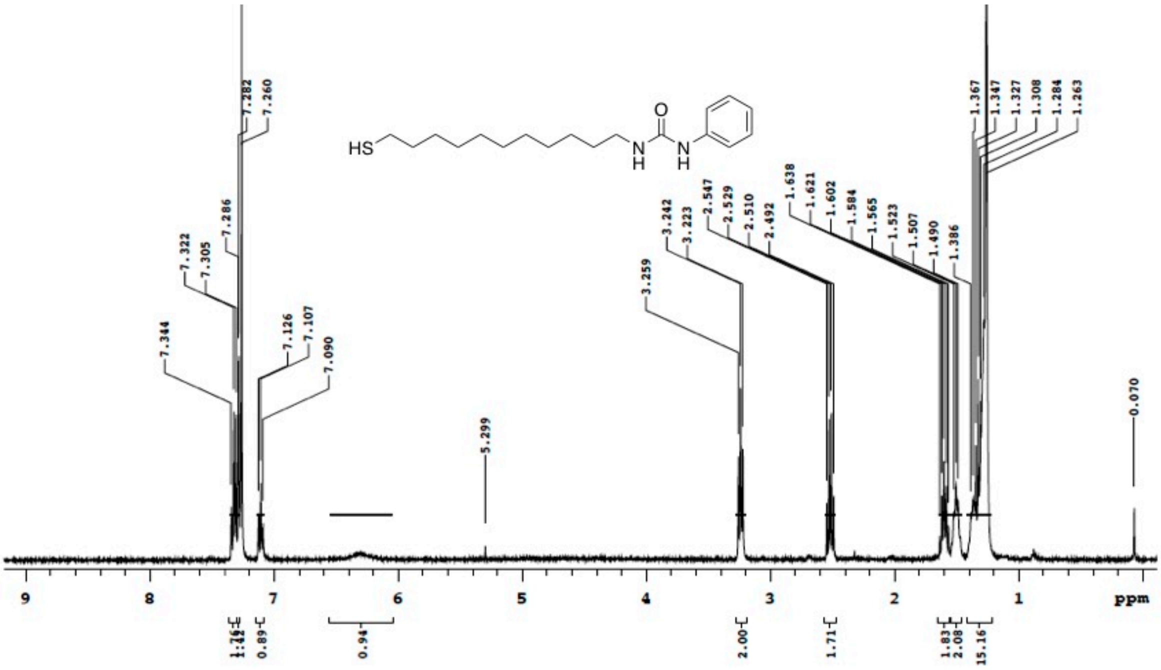

^{13}C NMR spectrum (100 MHz, $CDCl_3$) of **6.3**

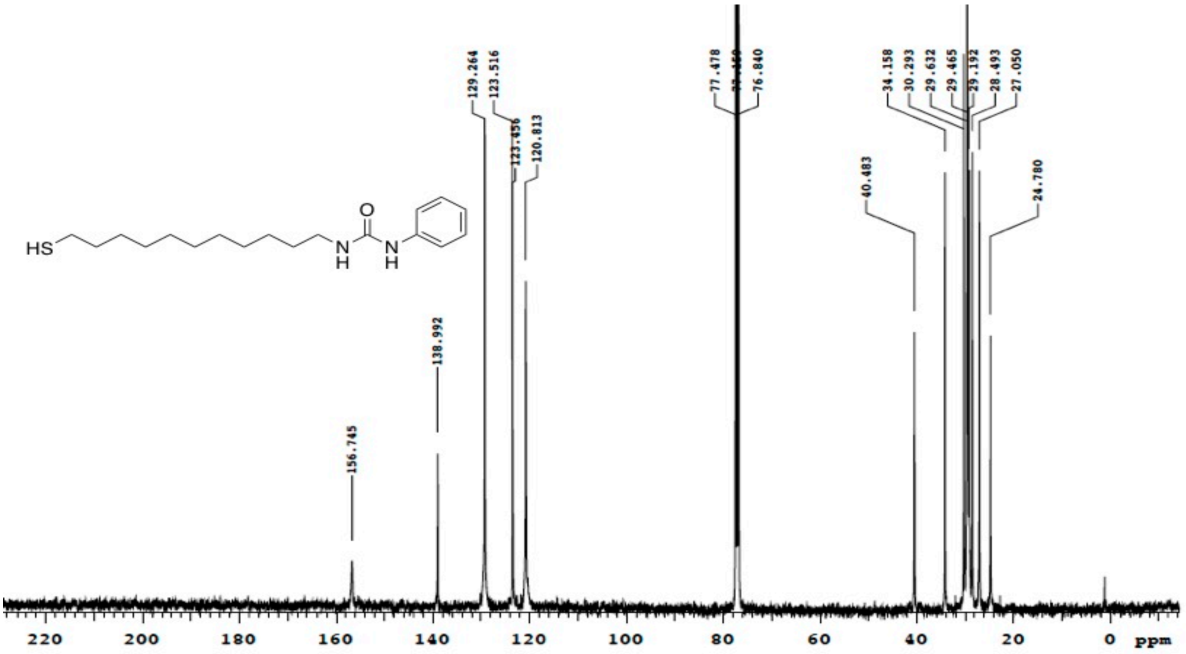

^{1}H NMR spectrum (400 MHz, $CDCl_3$) of thioester precursor to **6.4**

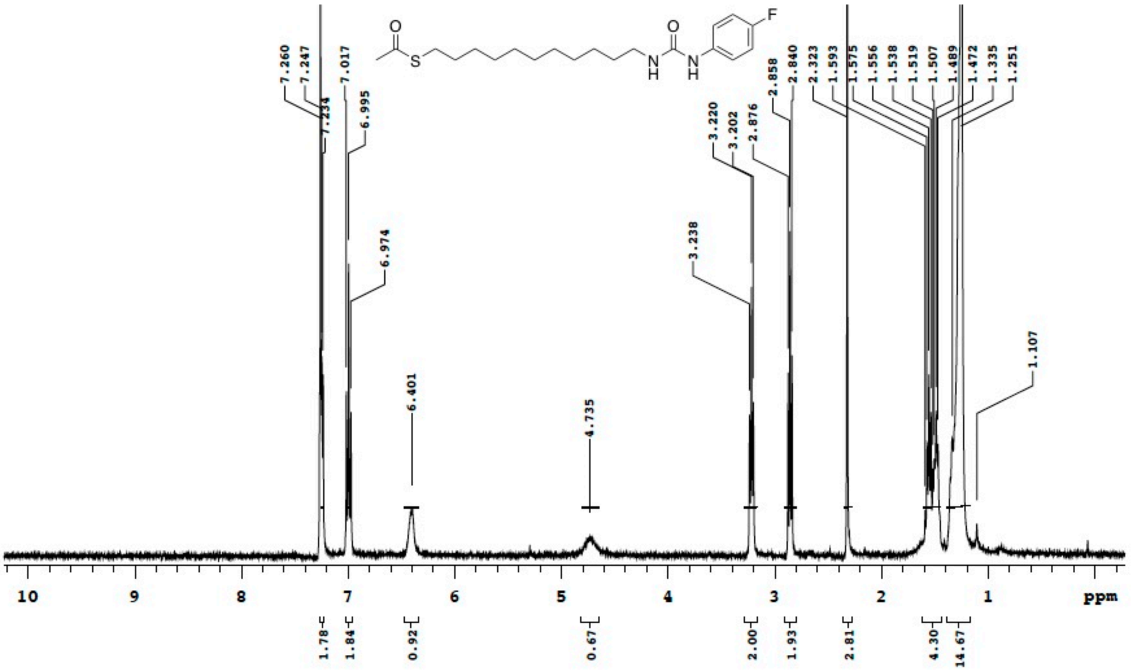

^{13}C NMR spectrum (100 MHz, $CDCl_3$) of thioester precursor to **6.4**

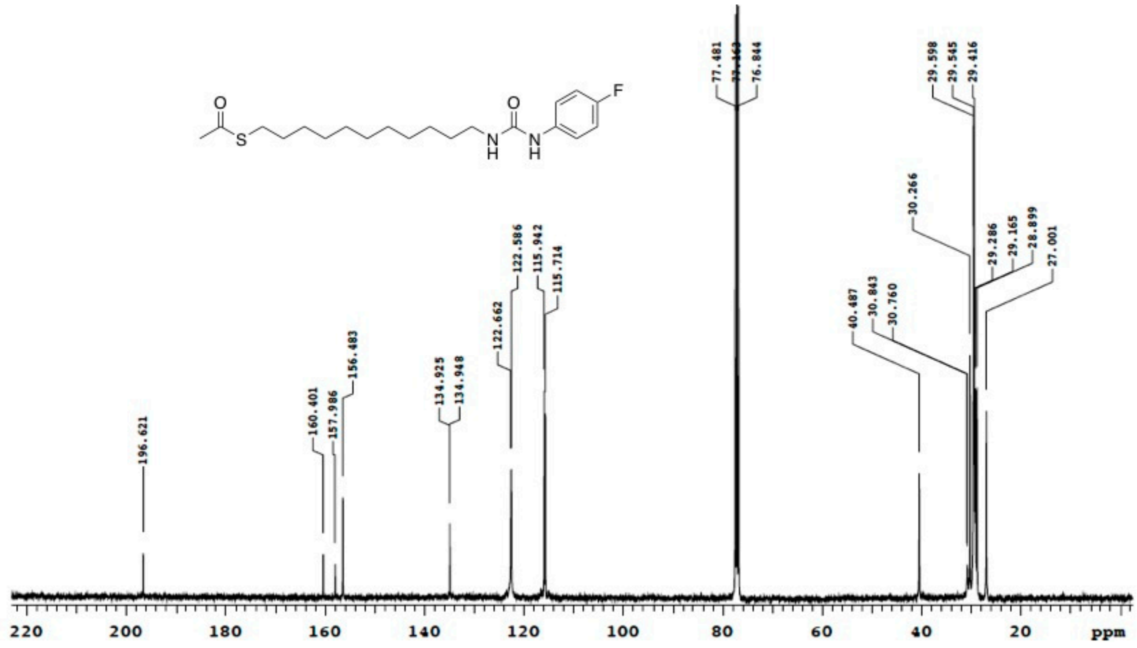

^{1}H NMR spectrum (400 MHz, $CDCl_3$) of **6.4**

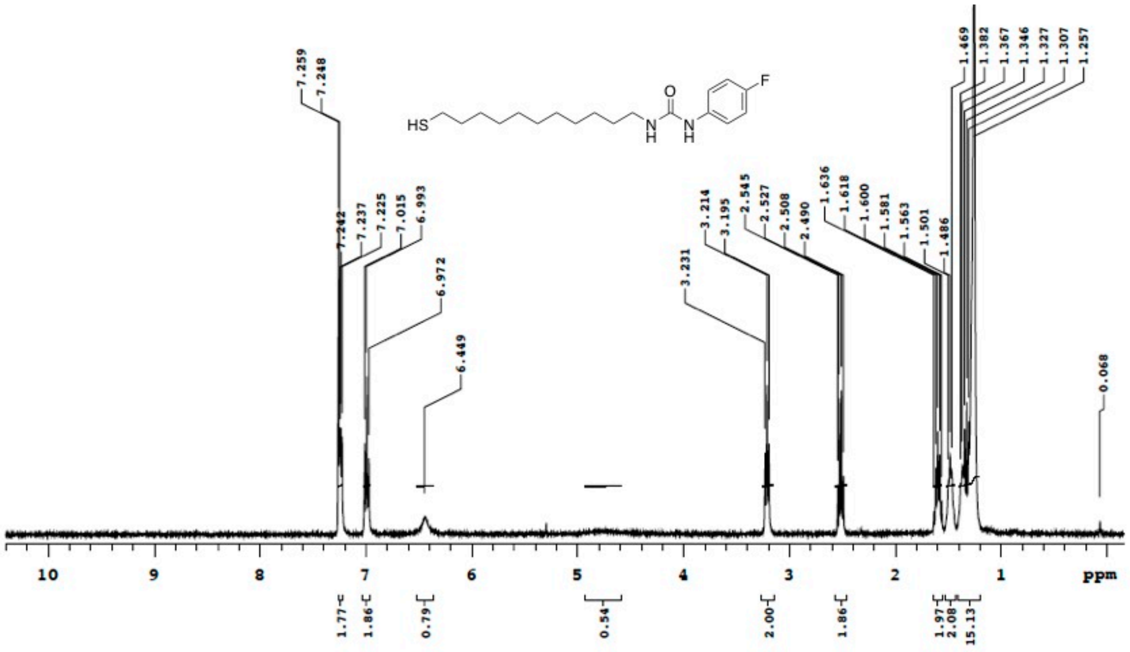

^{13}C NMR spectrum (100 MHz, $CDCl_3$) of **6.4**

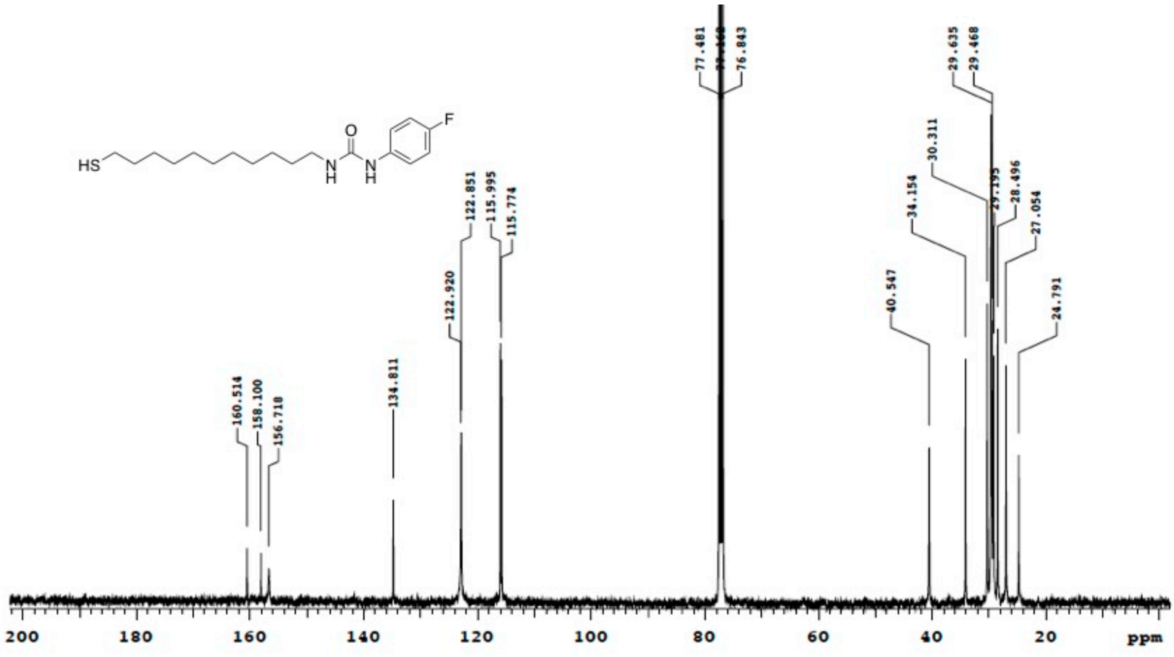

^{1}H NMR spectrum (400 MHz, $CDCl_3$) of **7.1**

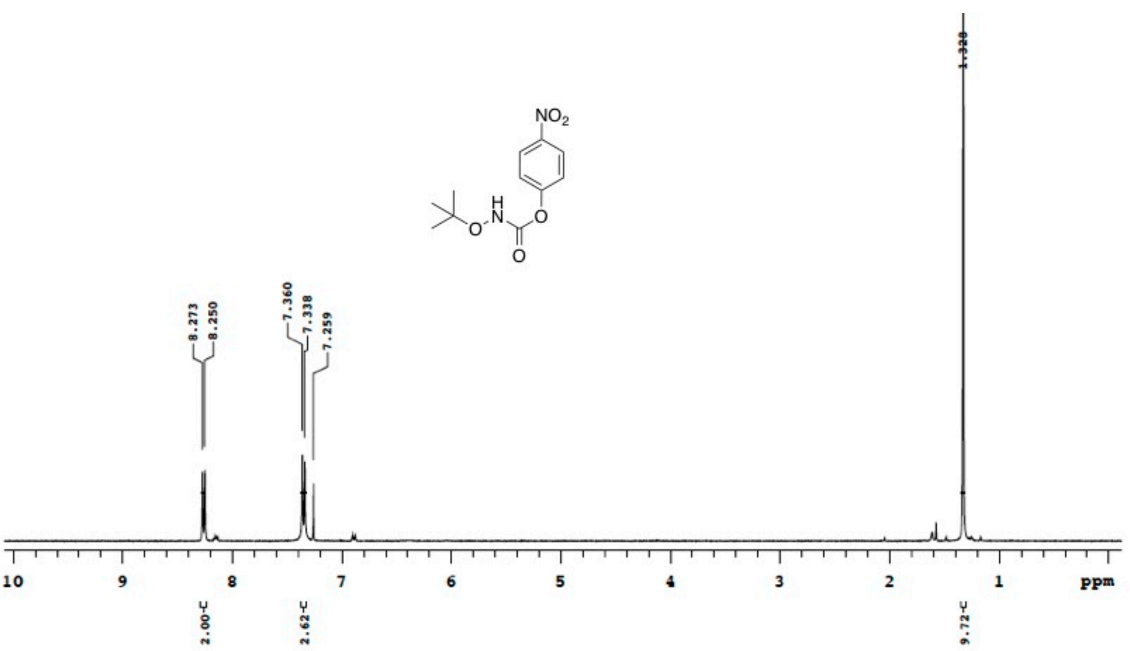

^{13}C NMR spectrum (100 MHz, $CDCl_3$) of **7.1**

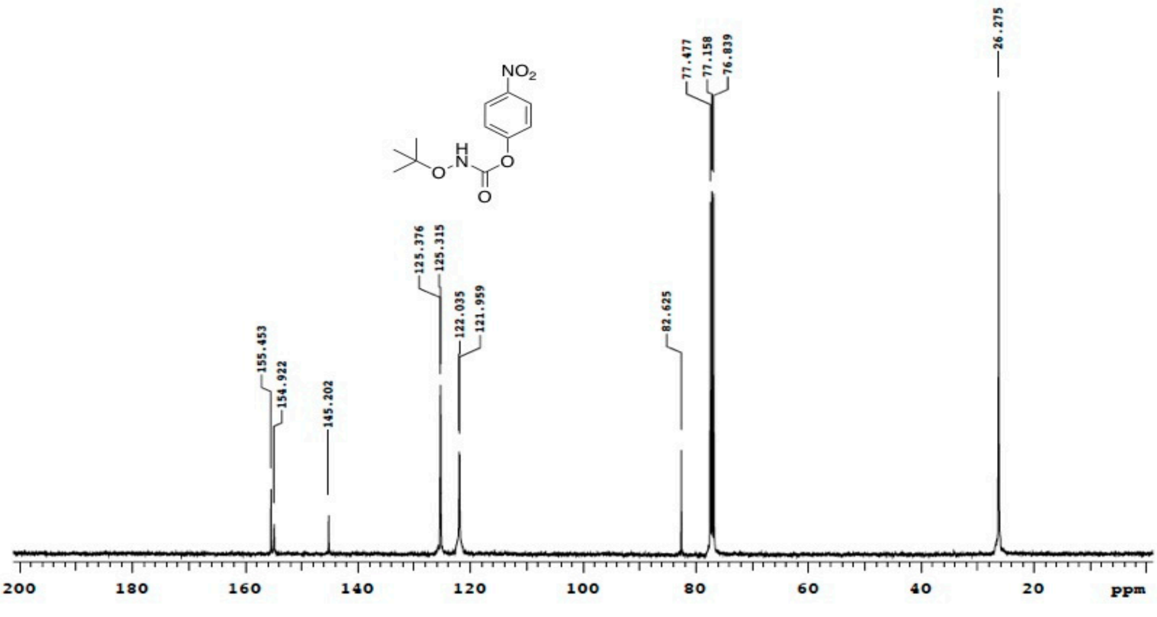

^{1}H NMR spectrum (400 MHz, $CDCl_3$) of **7.2**

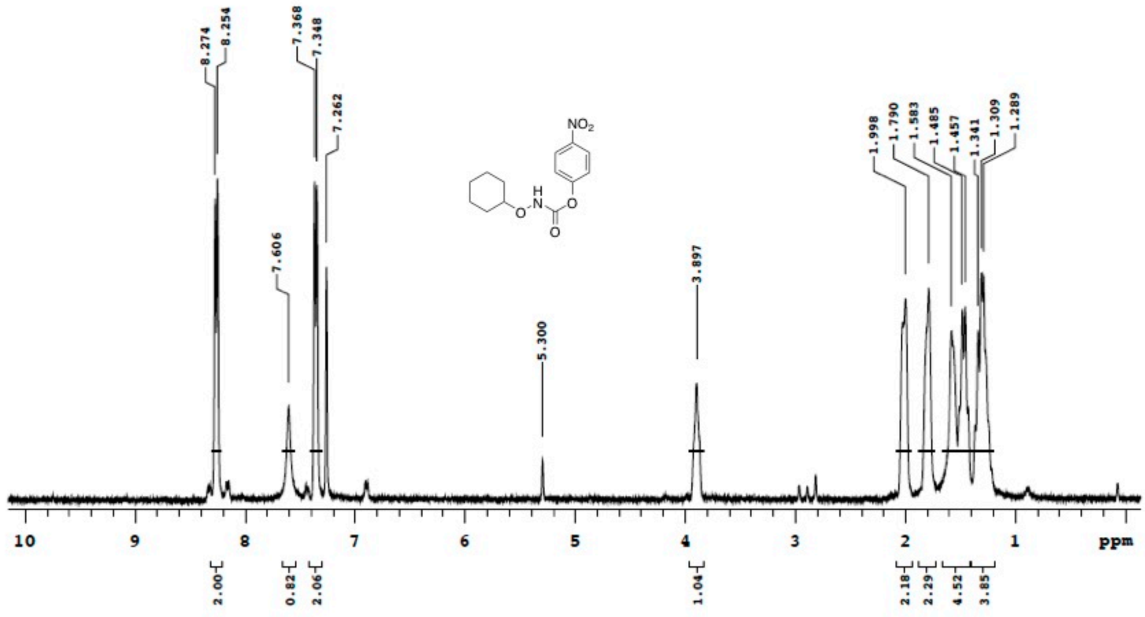

^{13}C NMR spectrum (100 MHz, $CDCl_3$) of **7.2**

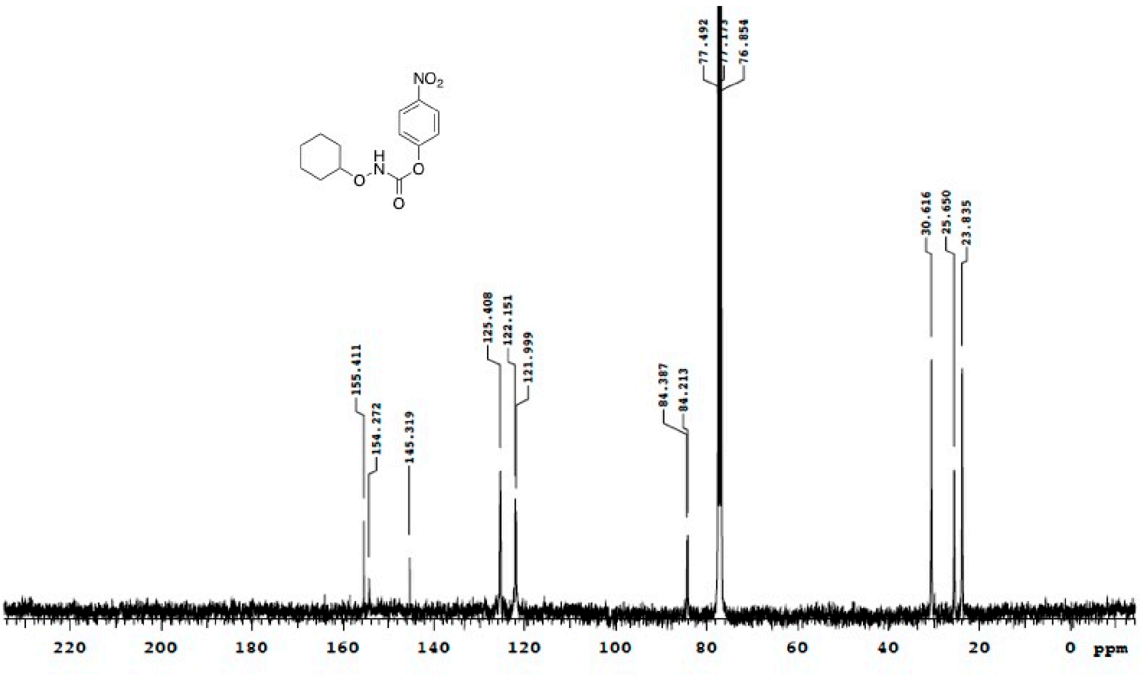

^{1}H NMR spectrum (400 MHz, $CDCl_3$) of **8.1**

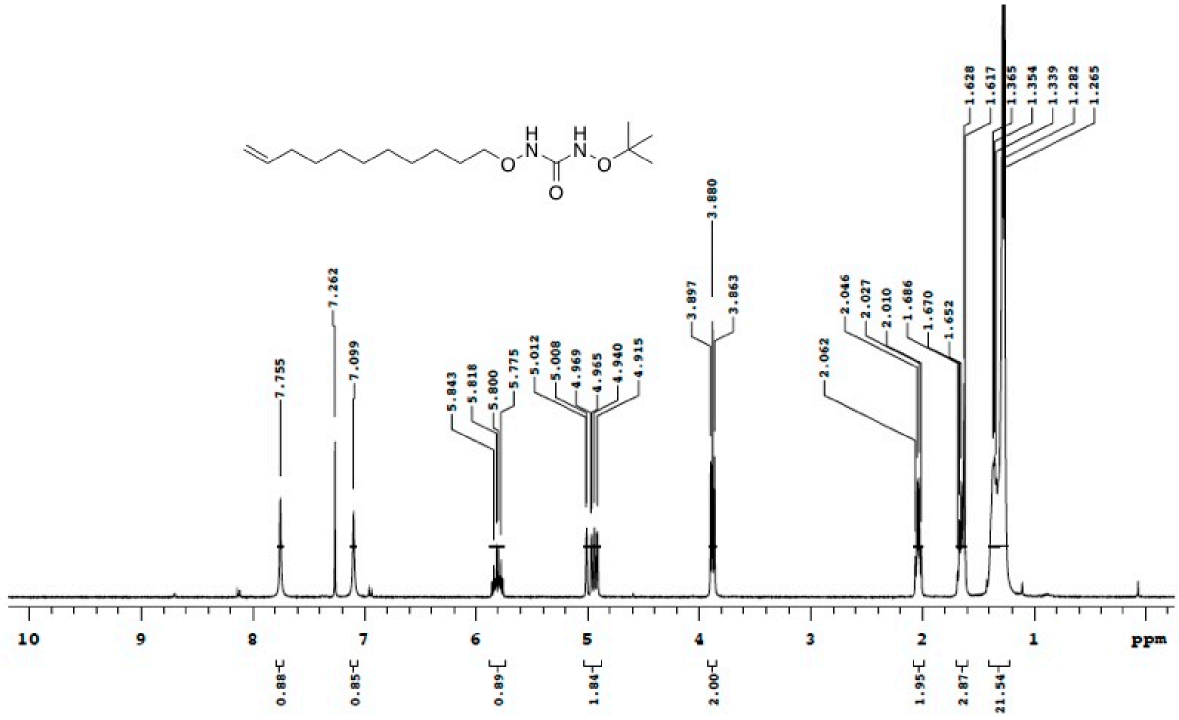

^{13}C NMR spectrum (100 MHz, $CDCl_3$) of **8.1**

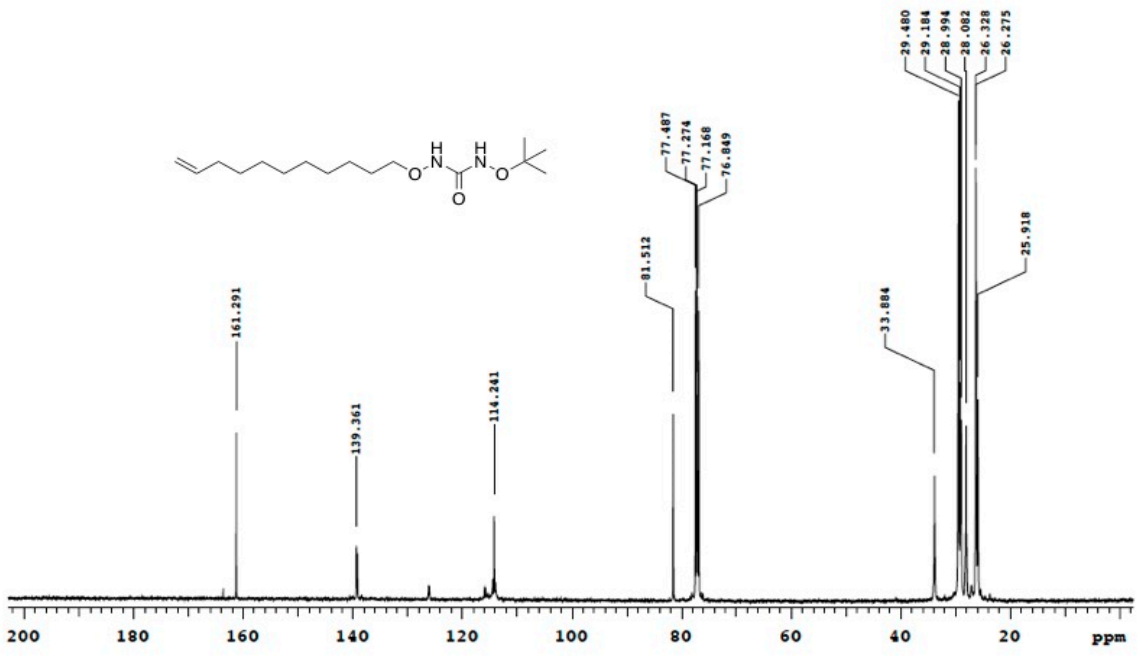

^{1}H NMR spectrum (400 MHz, $CDCl_3$) of **8.2**

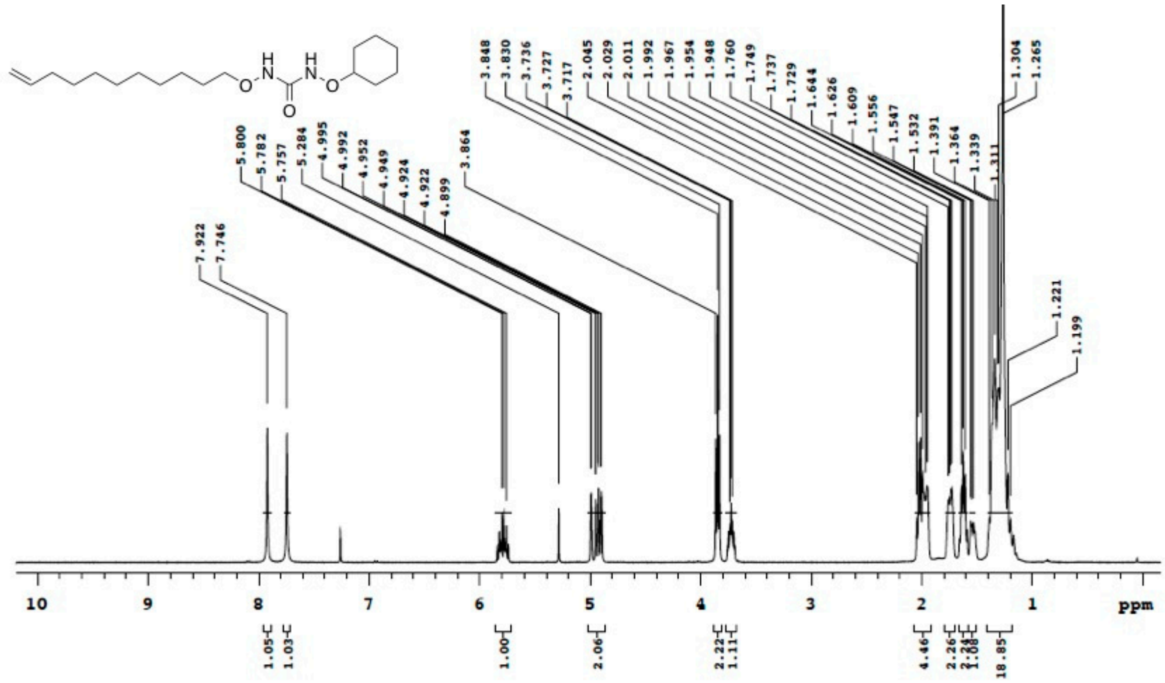

^{13}C NMR spectrum (100 MHz, $CDCl_3$) of **8.2**

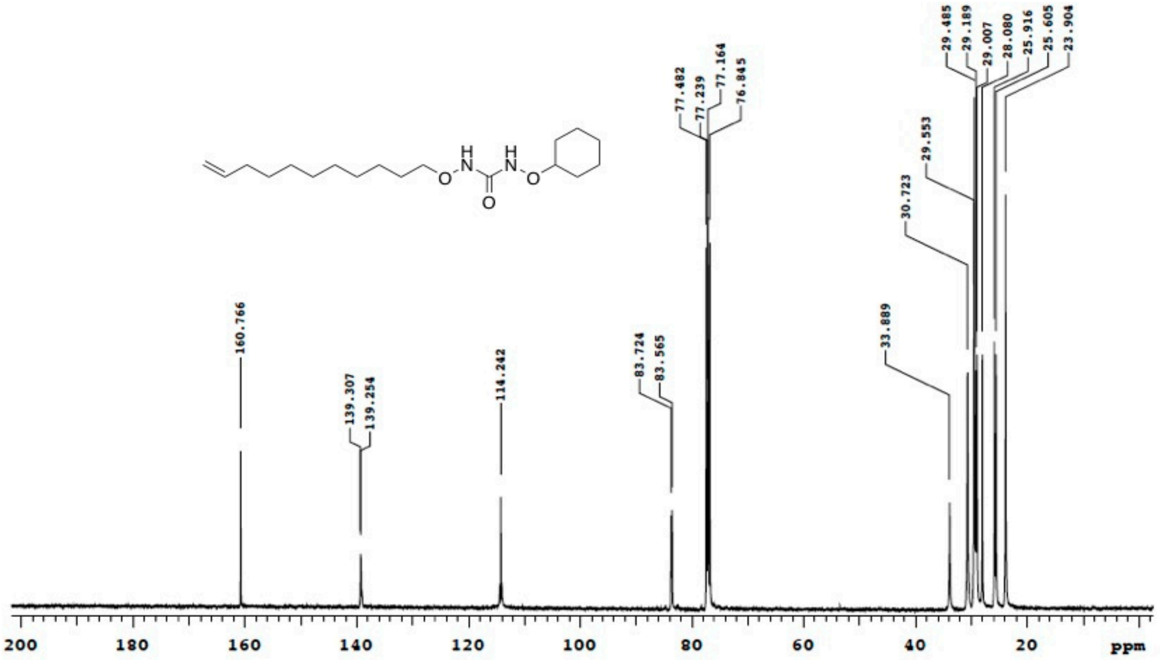

^{1}H NMR spectrum (400 MHz, $CDCl_3$) of thioester precursor to **9.1**

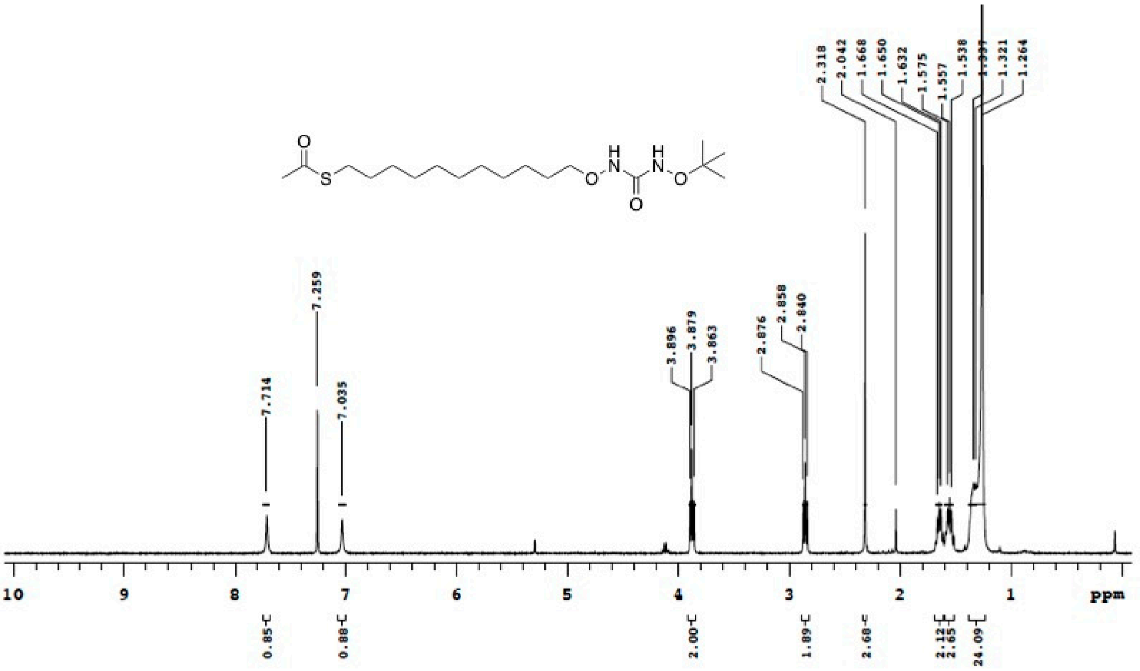

^{13}C NMR spectrum (100 MHz, $CDCl_3$) of thioester precursor to **9.1**

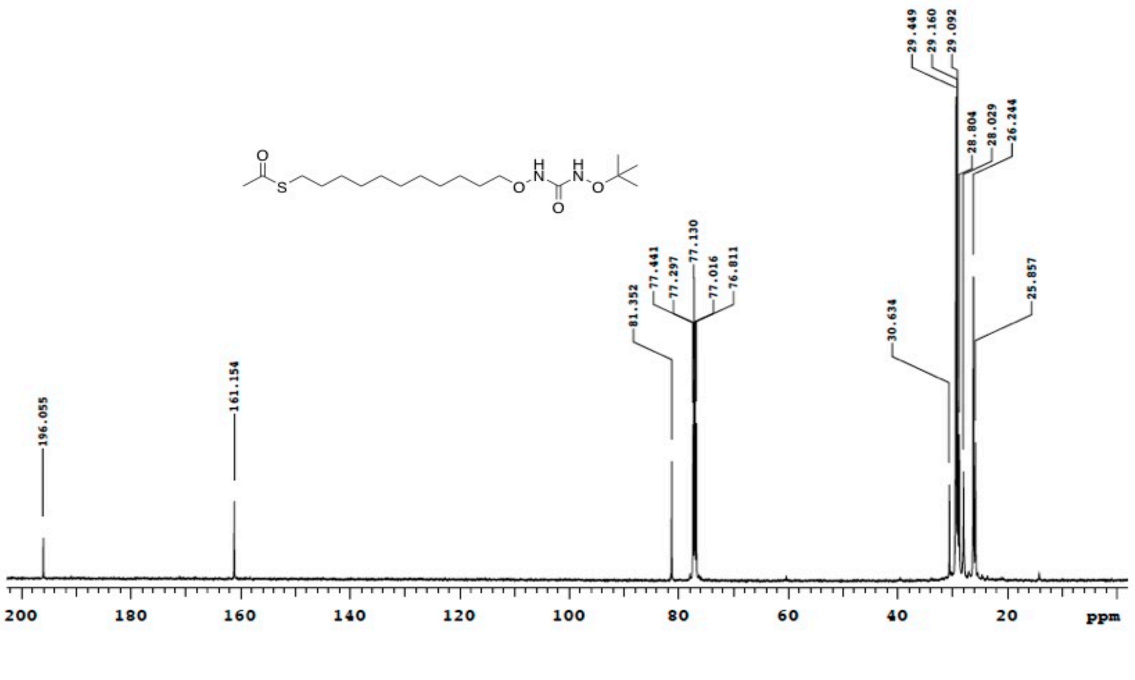

^{1}H NMR spectrum (400 MHz, $CDCl_3$) of **9.1**

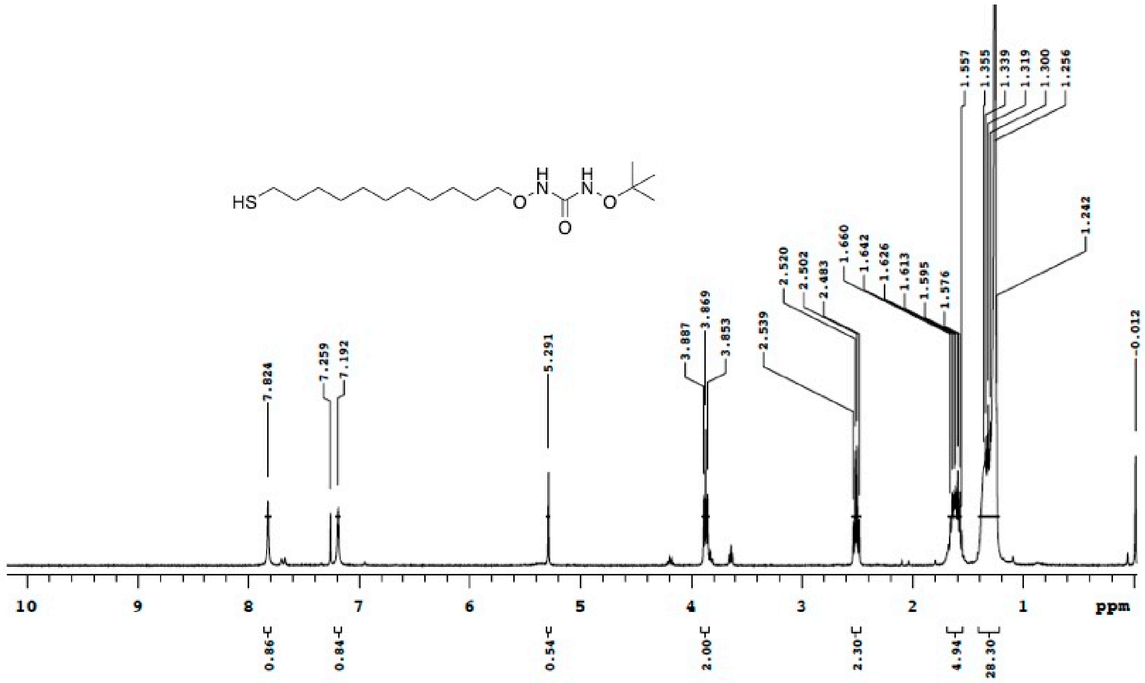

^{13}C NMR spectrum (100 MHz, $CDCl_3$) of **9.1**

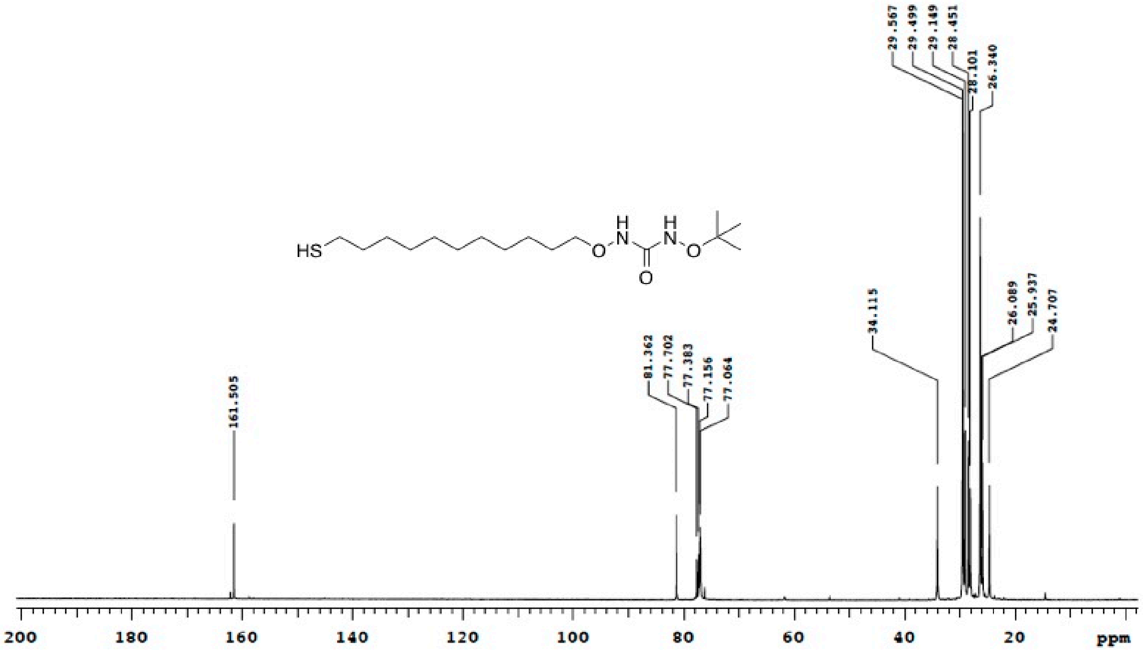

^{1}H NMR spectrum (400 MHz, $CDCl_3$) of thioester precursor to **9.2**

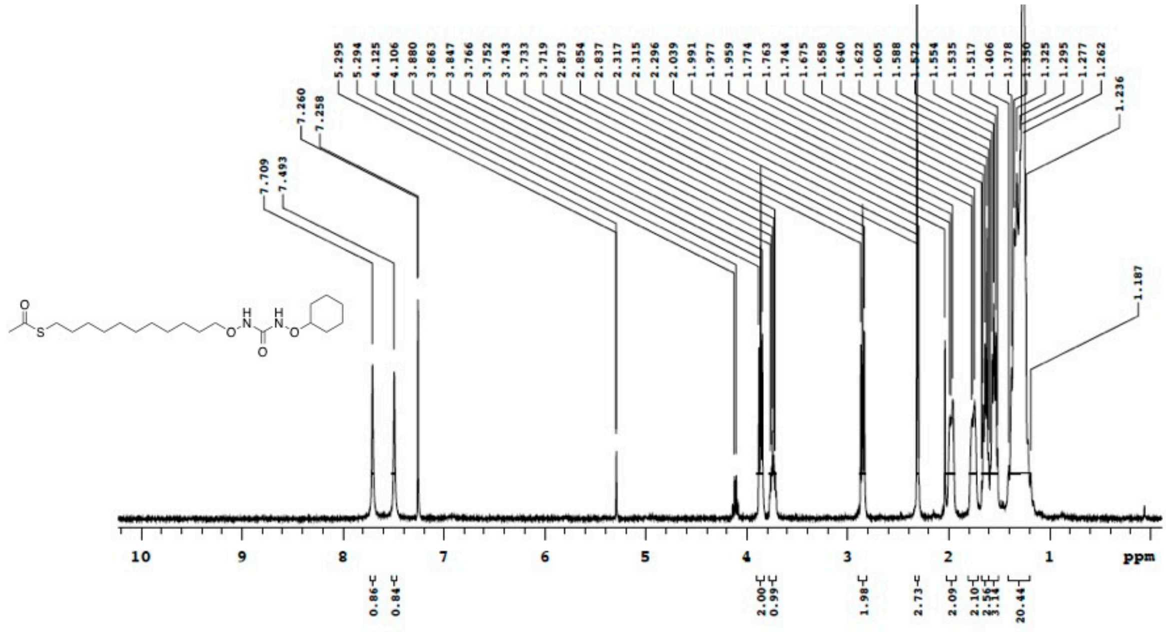

^{13}C NMR spectrum (100 MHz, $CDCl_3$) of thioester precursor to **9.2**

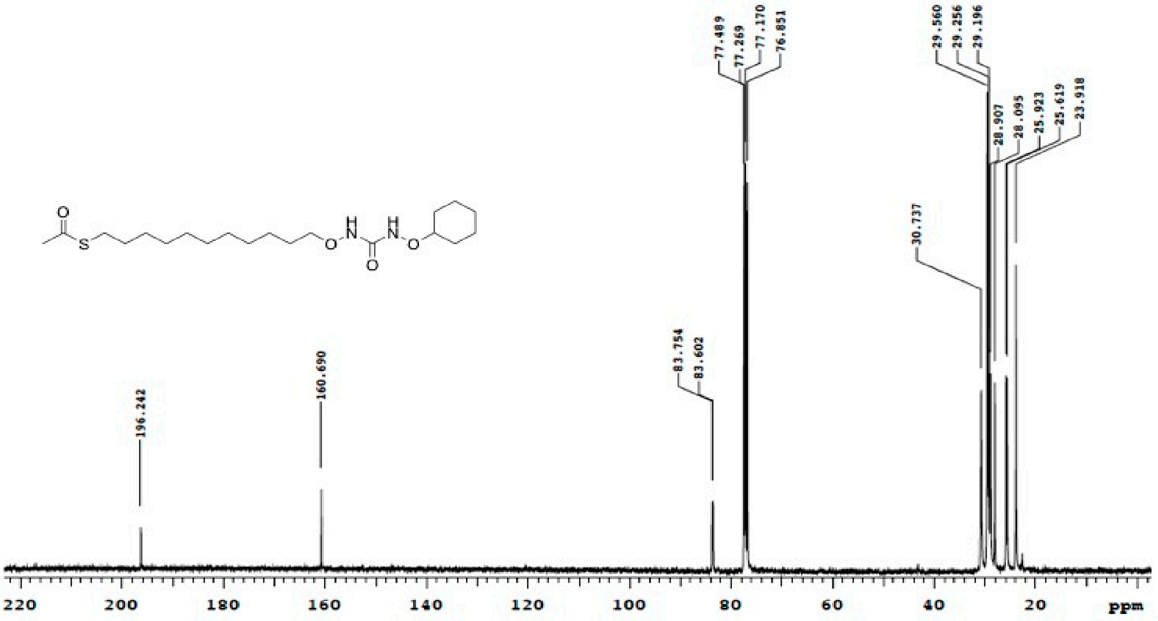

^{1}H NMR spectrum (400 MHz, $CDCl_3$) of **9.2**

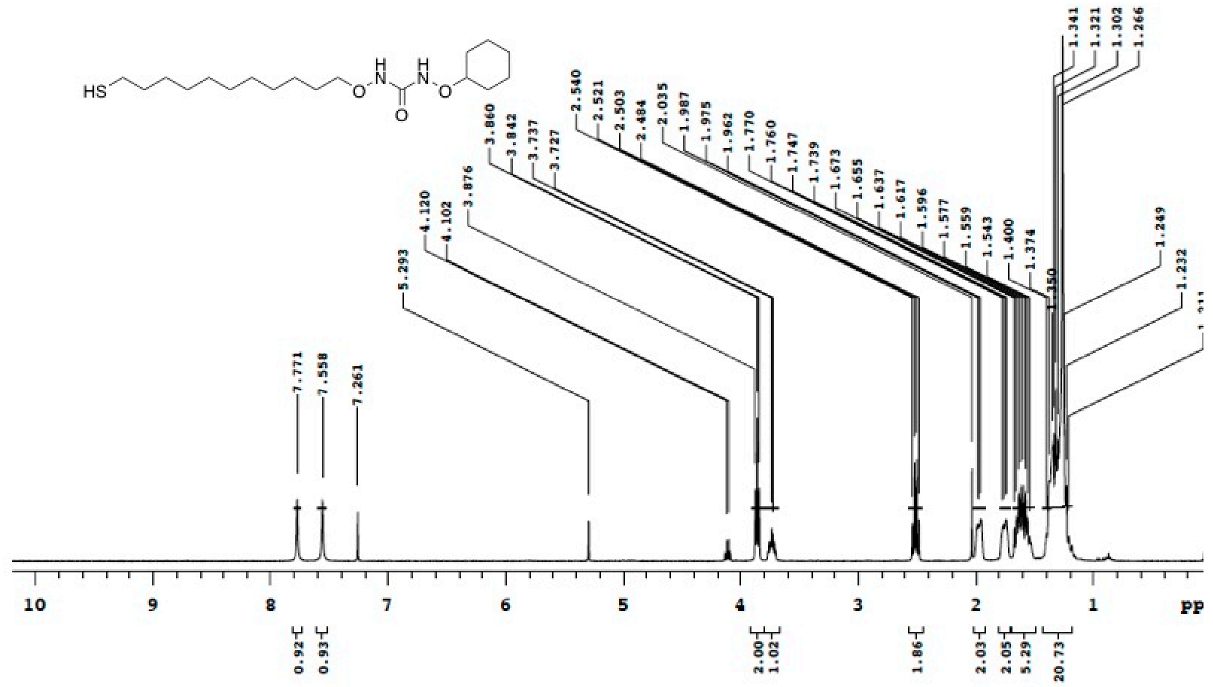

^{13}C NMR spectrum (100 MHz, $CDCl_3$) of **9.2**

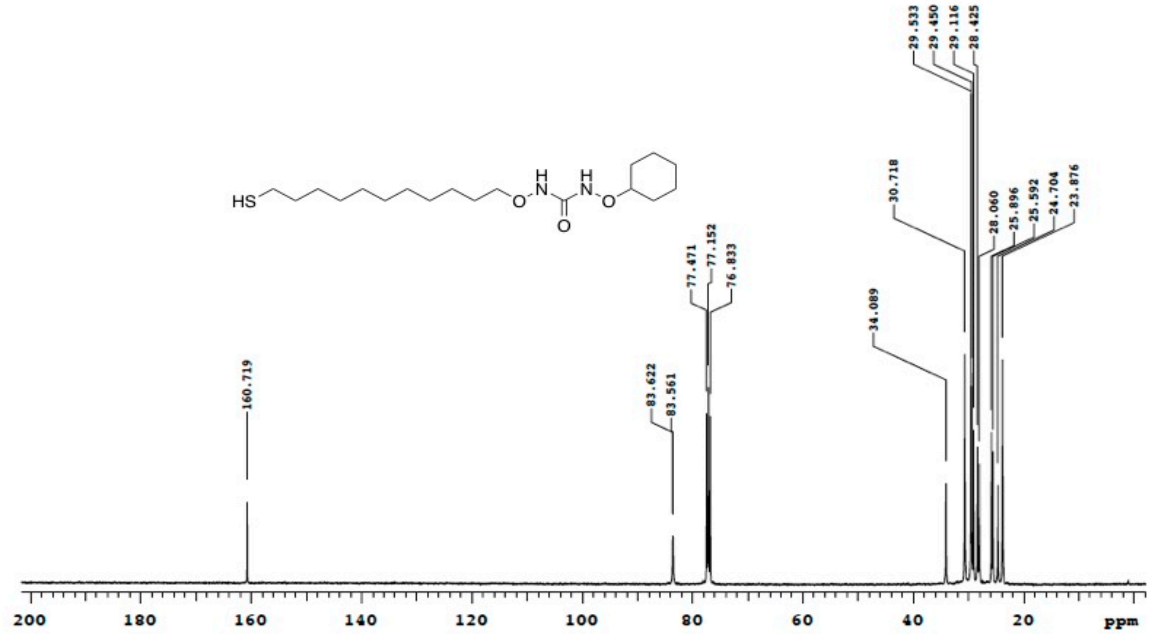

Supplement: Supplementary file 1 [file sensors-20-07024-s001.pdf]
